# Anatomy-aware, label-informed approach improves image registration for challenging datasets

**DOI:** 10.1371/journal.pone.0357448

**Published:** 2026-09-08

**Authors:** Rachel A. Roston, Nicholas J. Tustison, A. Murat Maga

**Affiliations:** 1 Center for Developmental Biology and Regenerative Medicine, Seattle Children’s Research Institute, Seattle, Washington, United States of America; 2 Department of Radiology and Medical Imaging, University of Virginia, Charlottesville, Virginia, United States of America; 3 Department of Pediatrics, University of Washington, Seattle, Washington, United States of America; Massachusetts General Hospital, UNITED STATES OF AMERICA

## Abstract

Image registration-based volumetric morphometrics have emerged as a valuable method for identifying subtle morphological differences in neuroimaging and other biomedical images. However, accurate registration out-of-the-box remains challenging when overt morphological phenotypes are present, limiting the application of registration-based morphometrics in developmental and comparative studies where overt phenotypic differences are common. A new label-informed image registration function developed in the ANTsX ecosystem provides an easy to use, generalizable solution for anatomy-aware registration of a wider diversity of morphological variation including many overt phenotypes. In this approach, segmentations (*i.e.*, labels) provide *a priori* regional correspondences that guide the registration, allowing morphological experts to define regions of correspondence based on biological concepts of homology (*e.g.,* tissue origin, gene expression patterns). Here we demonstrate the utility of this label-informed image registration approach for registering knockout mouse embryos with overt phenotypes which fail to register to a wildtype (normative) template image by traditional registration methods. Due to severe scoliosis, E15.5 *Gli2*^*-/-*^ mouse embryos exhibit a radical topological rearrangement of the internal organs; traditional intensity-only registration fails to accurately align the organs of knockout embryos with the normative template, limiting the interpretability of registration-based morphometric analyses. In contrast, label-informed image registration improved the correspondence of knockout subjects to the canonical template image, increasing the biological interpretability, power, and sensitivity of registration-derived morphometrics. All in all, label-informed image registration provides a flexible and customizable method to allow image registration in datasets for which registration-based morphometrics were previously unfeasible, unlocking new potential applications of registration-based morphometrics in developmental, comparative, and evolutionary studies.

## Introduction

Morphometrics–the mathematical and statistical study of form–is an essential part of studying biological variation [1,2]. Uncovering the origins and causes of morphological variation requires the ability to quantify phenotypic differences even when there are overt differences in morphology. Overt morphological differences include meristic differences (*i.e.,* differences in the number of repeated structures such as vertebrae), missing anatomical structures, or large-scale differences in the shapes of structures. Quantitative morphometrics provide important insights into the developmental, physiological, and evolutionary mechanisms that produce qualitative phenotypes [3]. Many, if not most, qualitative phenotypic differences begin as quantitative differences at earlier developmental timepoints or lower hierarchical levels of biological organization (*e.g.*, cell signaling, molecular interactions) [4].

Over the past century, comparative morphometrics has undergone multiple revolutions [5–8]. Advances in imaging and surface scanning, software, and computational methods have made it easier than ever to collect, analyze, and interpret morphometric data for a wide array of biological questions (*e.g.*, [9–14]). Numerous tools and methods have been developed to analyze diverse morphologies. In its earliest form, morphometrics involved linear measurements collected with calipers or rulers, angles measured with protractors, and calculated ratios [8]. In the late 20th century, 2D and 3D landmark-based geometric approaches to biological shape analysis gained popularity [5,7,8,15]. While GM provides spatial information not captured by linear measurements, landmarks provide only sparse sampling of overall morphology [5,8,16]. Technological innovations have also enabled other morphometric approaches to quantify shape differences from external outlines (*e.g.,* elliptical Fourier analysis), surfaces, point clouds, and more (*e.g.*, [10–12,14,17,18]). Outline and surface-based metrics may capture more morphological detail than landmarks due to denser sampling, but internal structures are often still missing from these analyses. These methods have been applied to wide variety of study subjects, from plant leaves to vertebrate skulls, and have been applied to a wide variety of biological questions from embryonic development to biological variation to evolution [8,19–22].

Volumetric morphometric approaches capture dense volumetric shape information that cannot be captured with landmark- or surface-based approaches [16,23]. In voxel-based morphometrics, image registration maps each voxel in a 2D (*e.g.,* photograph) or 3D image (*e.g.,* CT scan, MRI) to a template image, calculating the deformations required to superimpose the subject image with the template [16,23]. Transformed images or the deformation fields can then both be analyzed using statistical methods to measure differences in voxel values, volume, shape, and many other aspects of morphology [24]. Volumetric morphometrics are widely applied in human biomedical imaging contexts, especially in neuroimaging [25–28]. But, their application to a wider variety of research subjects and biological questions has remained relatively limited, in part due to limitations of image registration techniques.

### Image registration

“Classical” or traditional image registration approaches iteratively map one image to another image based on optimizing a similarity metric, usually related to voxel intensities (such as mean-squared error and mutual information), and outputs a regularized transformation that mathematically describes the mapping from one image into the space of another [29]. Registration approaches vary based on the transformation model, similarity metric, and optimization strategy employed. Registration generally proceeds in a hierarchical manner, starting with gross alignment of the images through linear transformations (*i.e.,* translation, rotation, scaling, and shear) and then fine-tuning image alignment with local transformations [16,30,31]. Performance can vary based on geometric similarity and intensity distribution, which is often determined by such considerations as regions of interest [31], inter- vs. intra-modality [32], and pathology vs. non-pathology [33,34]. In the case of overt morphological differences and multiple regions of interest, image registration is further complicated and often yields suboptimal results using traditional approaches [31].

Anatomy-aware registration approaches aim to include additional anatomical information in a registration pipeline to obtain better image alignment. For example, brain registration has historically been facilitated by masking out the skull (*i.e.*, “brain extraction) [35]. Similarly, modern approaches integrate deep learning (DL) by including a DL-based segmentation or feature extraction step into the registration pipeline or by incorporating anatomical/biomechanical parameters in the DL loss function. However, many existing DL-based anatomy-aware registration approaches are task- or population-specific, often due to training characteristics, limiting their generalizability [36–38].

Here we present a flexible and customizable anatomy-aware registration approach available as new functionality in the open-access, open-source ANTsX ecosystem [9,13,39]. Label-informed image registration allows the actual morphology to guide image registration according to the specific goals and questions of a study by using labels (*i.e.*, segmentations) to initialize and guide intensity-based image registration. Corresponding regions may be identified based on expert knowledge of developmental origins, gene expression, or other criteria relevant to a study, information that may not be readily apparent or contained within an image itself. Morphologists are therefore able to choose which features or regions should be considered homologous and compared based on biological concepts of homology (*e.g.*, [7,40–48]). The selection and weighting of labels can also allow customized selection of regions of interest to be prioritized in the registration. For example, a missing structure (*e.g*., a missing heart) can cause neighboring structures (*e.g*., lungs) to radically shift in position. While the missing structure itself cannot be registered, label-informed registration can support the registration of the neighboring structures despite their radical shift in position.

### Automated phenotyping of genetic knockouts

In this study, we aim to demonstrate how label-informed image registration can be used to improve image registration when overt morphological differences are present by applying it to the example of phenotypic analysis in a reverse genetic screen in mice. Collective and collaborative imaging efforts in mouse developmental-genetics and phenotyping has led to a growing abundance of knockout mouse image data, which necessitates high-throughput, computational pipelines for phenotype analysis [33,49,50]. Several registration-based analytical pipelines have been proposed to analyze and phenotype mouse knockout embryo images, but these approaches have not yet gained widespread application [31,34,50–54].

In the proposed analytical frameworks, knockout and wildtype embryos are imaged with diffusible iodine contrast-enhanced micro-CT or micro-MRI and the images are registered to a common template image [31, 34, 50–54]. The subject images may be registered to a population template [34] or to a normative template averaged only from wildtypes [31]. A population template is unique to the dataset, but a normative template allows all knockout lines to be registered to a single template; thus, the latter approach permits comparisons between knockout lines, between studies [31]. It also speeds up analyses by eliminating the time-intensive step of generating an average template image for each knockout dataset [31]. The output of the registration—both transforms and transformed images—are then analyzed statistically (*e.g.,* using tensor-based morphometry, TBM, or other methods) to identify phenotypic differences between knockout and wildtype embryos [31,34,50–54].

Knockout phenotypes often include overt phenotypic differences—such as missing structures or large-scale differences in embryo geometry—that may impede both traditional and DL-based approaches to image registration. Incomplete penetrance and variable expression of phenotypes in reverse genetic screens mean that optimal parameters for one subject may not apply to another subject with the same genetic mutation [31]. Also, the whole-body surveys preferred for phenotype discovery require simultaneous registration of multiple anatomical structures, each of which can vary greatly in size, shape, position, and orientation, and in their topological inter-relationships. Selecting registration parameters often requires balancing the accuracy of registration in one region with sacrificing accuracy in another region [31]. As a result, traditional registration approaches may not result in accurate mapping between images, increasing morphometric error and diminishing the interpretability of morphometric results. Mutations also often result in prenatal lethality and limited viability, resulting in relatively small sample sizes in studies [33,50]. The combination of small sample sizes and highly variable morphological phenotypes limit the training data available for anatomy-aware DL-based registration approaches. Challenges with image registration have consequently limited the range of knockout phenotypes that can be analyzed using proposed phenotypic analysis pipelines.

**Fig 1:**
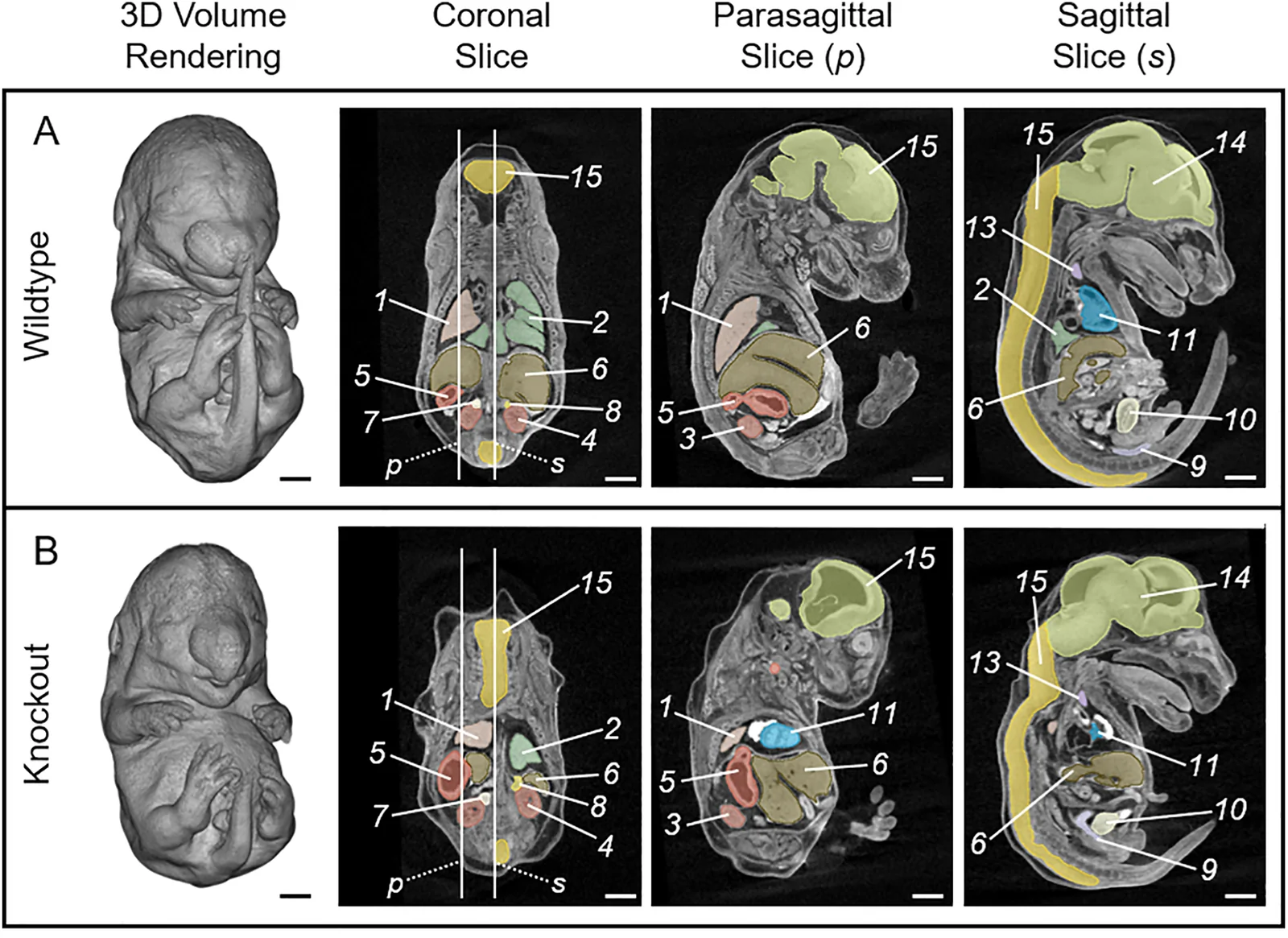
Representative diceCT scans showing organ topology in (A) a representative wildtype (Gli2^+/+^; Scan_0093) and (B) a representative knockout (Gli2^-/-^; Scan_0105) subject. Vertical lines in the coronal slice show the locations of the parasagittal (*p*) and sagittal (*s*) slices for each subject. Scale bar = 1 cm. Numbers indicate organ label: *1 left lung, 2 right lung, 3 left kidney, 4 right kidney, 5 stomach, 6 liver, 7 left adrenal gland, 8 right adrenal gland, 9 rectum, 10 bladder, 11 heart ventricles, 12 left thymic rudiment, 13 right thymic rudiment, 14 brain, 15 spinal cord.*

As a test case for using label-informed image registration for morphometric analysis of subjects with overt phenotypic differences, we selected a genetic knock-out mouse line that has failed to accurately register to a wildtype-based template image using existing intensity-only registration methods. This has impeded the application of registration-based morphometric pipelines for phenotype discovery described by Wong et al. (2014), Dickinson et al. (2016), and Horner et al. (2021) [31,34,50]. GLI2 is a well-studied transcription factor involved in transduction in the sonic hedgehog (SHH) signaling pathway [55]. Knocking out *Gli2* causes multiple documented overt phenotypes across the whole embryo, including developmental anomalies in the brain, face, spinal cord, vertebral column, heart, lungs, digestive system, and urogenital system [55] (Fig 1). Missing and misshapen vertebrae have previously been described in *Gli2*^*-/-*^ mouse embryos [55,56] but the effects of these defects on overall geometry of the vertebral column and morphometrics of the embryo have not been previously studied. Additionally, *Gli2* knockout embryos are also suspected to have several quantitative phenotypes, such as a reduction in bladder volume and alterations in liver shape, but these have not previously been documented using volumetric morphometrics.

Here, we show that label-informed image registration aligned *Gli2* knockout embryos with the normative, wildtype-based template image more accurately than traditional intensity-only registration. This improved alignment increased the power, sensitivity, and interpretability of downstream statistical analyses. We demonstrate that TBM using label-informed image registration captured both overt, gross morphological differences and subtle, quantitative differences and that principal component analysis (PCA) on transforms generated by label-informed image registration also reflected observable, overt morphological differences that intensity-only registration failed to capture. Together, these findings suggest that label-informed image registration can broaden the application of registration-based morphometrics, including in contexts in which the presence of overt morphological differences was previously prohibitive.

## Methods

### Label-informed image registration

While **antsRegistration** remains the core framework for intensity-based registration, the recently added functionality of **ANTsR::labelImageRegistration()** in R (ANTsR) and **ants.label_image_registration** in Python (ANTsPy) leverages this inherent flexibility to provide a specialized workflow for intensity-based registration in cases where expert-defined or automated segmentations are available. This is particularly relevant given the increasing availability of deep learning-based segmentation tools.

Internal preprocessing involves converting input categorical label maps into paired continuous representations (*e.g.*, smoothed binary images). The initial global alignment is determined using the centers of mass and can incorporate various linear transforms. Alternatively, a pre-generated linear transform from a previous **antsRegistration** run can be provided.

In contrast to traditional scenarios where **antsRegistration** optimizes similarity based on voxel intensity (e.g., neighborhood cross-correlation), **labelImageRegistration** constructs a cost function based on the spatial overlap of corresponding labels. Specifically, the paired sets of continuous images are used to optimize deformable alignment in a pairwise fashion using the Mean Square Difference as the similarity metric. The total cost function, $E_{total}$, is defined as the weighted (user-defined) combination of these individual label metrics, with the optional inclusion of intensity-based metrics to further refine the alignment:


$$\begin{array}{c} E_{total}=\sum_{i=\text{1}}^{N}w_{i}\cdot M_{label,i}+w_{intensity}\cdot M_{intensity} \end{array}$$

where *N* is the number of labels, $w_{label,i}$ is the relative weighting of label images, $w_{intensity}$ is the relative weighting of the optional intensity-based pairwise image similarity calculation,

and $M_{label,i}$ and $M_{intensity}$ represent the metrics for the *i*-th label and the image intensities, respectively.

Using **labelImageRegistration** is not the same as using **antsRegistration** with a mask; while a mask tells the algorithm where to look, the labels tell the algorithm exactly what to align and where to move it. Using **antsRegistration** with a mask defines a window where the similarity metric is calculated. If the anatomy inside that window is relatively homogeneous or the initial alignment is poor, the optimizer lacks the ‘signal’ to pull corresponding structures together. In **labelImageRegistration**, the categorical labels are converted into continuous representations (like probability maps). This constructs an explicit cost function based on spatial overlap. In other words, the boundaries of the labels are effectively actively driving the moving anatomy toward the target anatomy. While masked registration requires dilating the mask to capture edge information at the boundaries, label-informed registration inherently focuses on these boundaries because it optimizes the overlap of the label volumes themselves.

### Embryo collection and micro-CT

**Fig 2:**
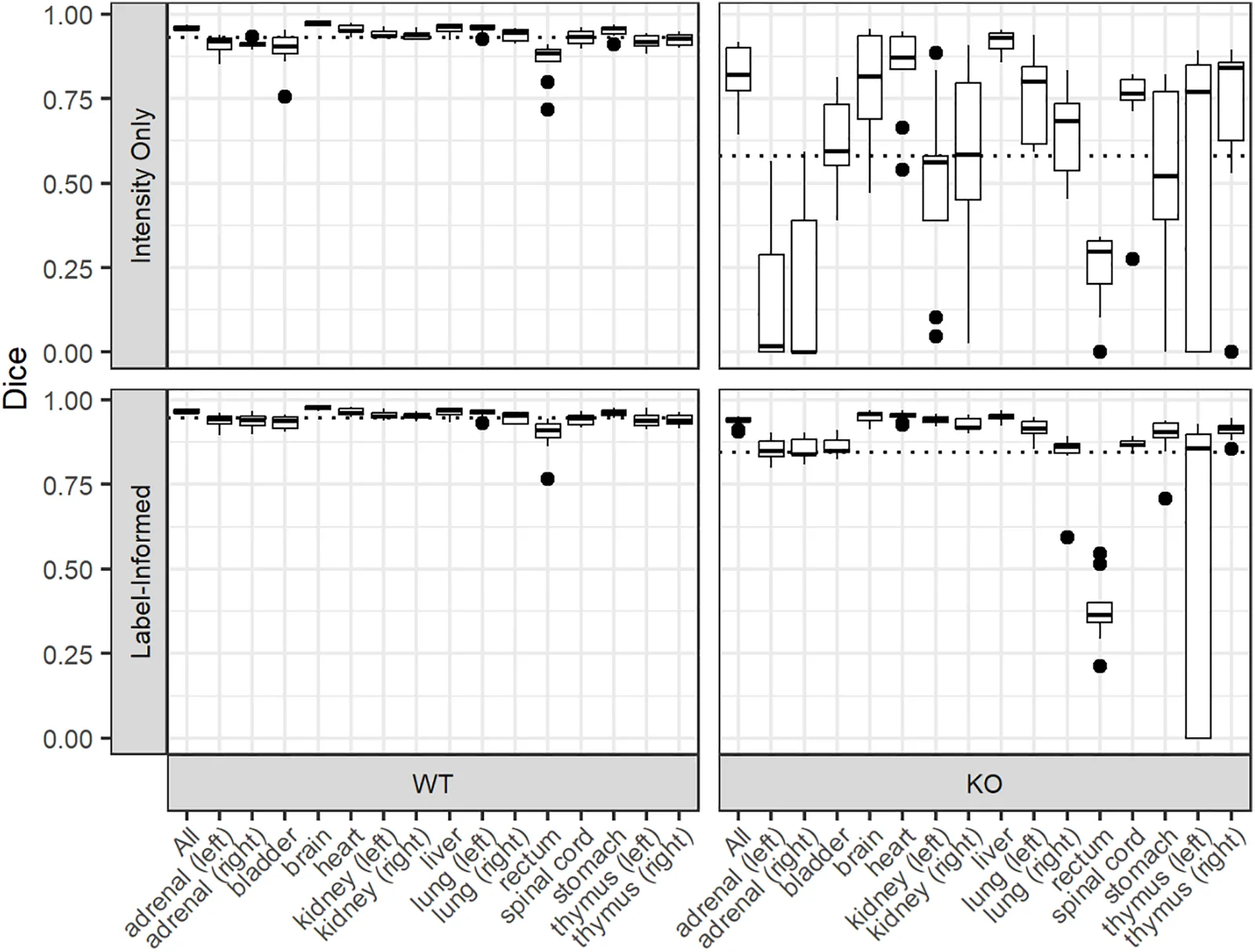
Mean overlap (Dice statistic) for (top) intensity-only registration-based organ labels and organ labels that were manually segmented by a morphology expert and (bottom) label-intensity registration-based organ labels and manually segmented organ labels. For wildtype embryos (WT, left), both registration approaches resulted in relatively high Dice statistics, most of which were above 0.80, indicating relatively good alignment of wildtype subject images with the template image. For knockout embryos (KO, right), intensity-only registration resulted in lower and more variable Dice statistics, indicating that intensity-only registration did not consistently align organs in knockout subjects to the organs in the template image; label-intensity registration increased the Dice statistics for knockout subjects for most organs and reduced their variability, indicating improved overall alignment of knockout subjects with the template image. Horizontal dotted line indicates the mean Dice statistic for each registration approach and genotype.

**Fig 3:**
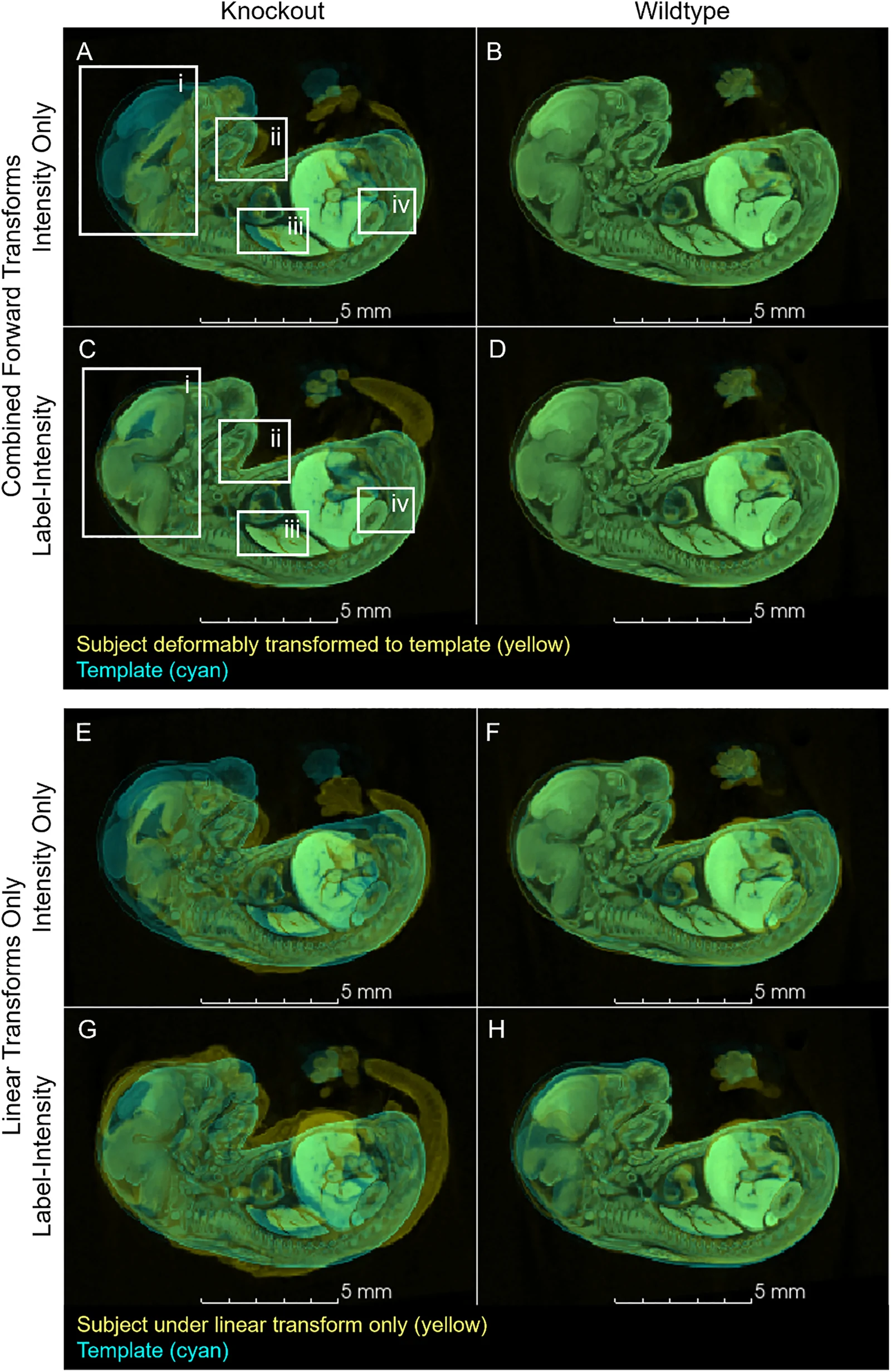
Intensity-only and label-intensity registration results for a representative knockout (*Gli2*^*-/-*^; Scan_0105) and a representative wildtype subject (*Gli2*^*+/+*^, Scan_0093) (yellow) overlaid over the template image (cyan). (A-D) Subjects transformed to template space using combined forward transforms. (E-H) Subjects under the initial linear transform only. For the knockout subject, intensity-only registration (A) resulted in a poor overlay of the brain (i), lower jaw (ii), lung (iii), kidney (iv), and other internal organs (not shown). Label-intensity registration (C) resulted in a more accurate overlay of the transformed subject image over the template image. In wildtype subjects, both registration approaches produce nearly identical results (B, D). The scaling associated with the initial linear transforms differ between registration approaches in both knockout and wildtype subjects (E-H), but the difference in scaling is much more apparent in knockouts (E, G). This representative wildtype subject did not contribute to the template image. In the images shown, the limbs and tail did not align accurately with the templateand were not the focus of this study because they vary randomly in positioning.

Nine *Gli2*^*+/+*^ and nine *Gli2*^*-/-*^ mouse embryos were collected on embryonic day 15.5 (E15.5) by S. Gombart and E. Vincent at Seattle Children’s Research Institute in accordance with IACUC protocol 00030. The 18 subjects (9 wildtype and 9 knockout) used in this study were a subset of 295 embryos of multiple knockout strains and genotypes collected and scanned for a reverse genetic screen. *Gli2* was selected for the present study because *Gli2*^*-/-*^ embryos have a severe, overt phenotype that failed to register accurately to a wildtype template image using existing intensity image registration methods (Figs 1–3).

Subjects were fixed overnight in 4% paraformaldehyde solution (PFA) and stored in PBS at 4 °C until preparation for scanning. To stabilize the soft tissues, subjects were embedded in hydrogel following Wong et al. (2013) and Dickinson et al. (2016) [50,57]. To summarize, hydrogel solution containing 4 °C 4% PFA (16% PFA in aqueous solution, VWR), 4 °C 4% acrylamide (Fisher Scientific), 4 °C 0.05% bis-acrylamide (Fisher Scientific), 0.25% VA-044 (FUJIFILM Wako Chemicals), 0.05% saponin (Sigma Life Science), and 4 °C PBS was mixed on ice and frozen in ~12-mL aliquots. In preparation for scanning, fixed specimens were transferred from PBS to liquid (thawed) hydrogel solution. After 70–74 hours of incubation at 4 °C, hydrogel was polymerized at 37 °C for three hours and excess hydrogel was carefully removed from the outside of the specimens. The specimens were stained in 0.1 N iodine solution (Sigma-Aldrich) for 24 hours. After 24 hours, specimens were washed in room temperature PBS twice to remove excess iodine from the outside of the subjects and embedded in 1% agarose to stabilize them for microCT scanning. 3D scanning of subjects was conducted at the SCRI MicroCT Imaging Facility (*RRID:SCR_024678*), using Bruker Skyscan 1272 microCT. Specimens were scanned with isotropic spacing of 9 um^3^ (frame averaging = 5) or 18 um^3^ (frame averaging = 3), a 1-mm aluminum filter, and vertical rotation of 180 degrees. DiceCT images acquired at 9um were resampled to 18um^3^ spacing prior to further analysis using the batch mode in the SkyscanRecontImport module from SlicerMorph [12].

### Template image

The template image for this study was generated by averaging thirty-five wildtype micro-CT volumes from eight inbred and outbred knockout mouse lines (Table 1). The most common strain backgrounds were C57BL/6J and C57BL/6NCrl. First, all subjects were rigidly aligned to a common orientation that is congruent with the standard anatomical planes. An initial linear average of the rigidly-aligned subjects was used to initialize the template building process. To build the template, all subjects were aligned with the initial linear average using both linear and deformable registration. Then, a new template image was created by averaging all the subjects in the new space and applying filters (*e.g.*, sharpening and smoothing). The process then repeated iteratively using the template image calculated from the previous iteration. A total of three iterations was used to obtain an anatomically correct template from the selected wildtype subjects. More information about ANTs template building process can be found in the ANTsX Github documentation [9] (https://github.com/ANTsX/ANTs/blob/master/Scripts/antsMultivariateTemplateConstruction2.sh).

**Table 1:** Knock-out lines and strain backgrounds used to build the normative (wildtype) template image.

Knockout Line	Strain Background	Number (n) Used in Building the Template Image
*Gli1*^tm2Alj^/J	Outbred: 35.3–41.2% 129S	5
*Gli2*^tm6Alj^/J	Outbred: 89.1–91.7% C57BL/6J	4
*Gli3*^tm1Alj^/J	Outbred: 89.1–91.7% C57BL/6J	1
*Sptan1*^em1(IMPC)Mbp^/Mmucd	Close to inbred: C57BL/6NCrl	9
*Tbx1*^em1(IMPC)Mbp^/Mmucd	Close to inbred: C57BL/6NCrl	8
*Tcf21*^em1(IMPC)Mbp^/Mmucd	Close to inbred: C57BL/6NCrl	4
*Ttc21b* ^aln^	Close to inbred: B6.A(FVB)	1
*Vangl2*^em1(IMPC)Mbp^/Mmucd	Close to inbred: C57BL/6NCrl	3

### Segmentation

Fifteen organ labels (*i.e.,* segments) were selected by combining labels used in the published IMPC atlas and MEMOS [53,58]. Segments labeled the following organs: brain, spinal cord, 2 thymic rudiments, 2 heart ventricles, 2 lungs, liver, stomach, 2 kidneys, 2 adrenal glands, bladder, rectum. Manual segmentation of individual organs and the whole subject mask was conducted using the segmentation tools in 3D Slicer and SlicerMorph [12,59,60].

### Registration

Prior to this study, intensity images were downsampled from 18 um^3^ to 60 um^3^ spacing to allow data-sharing and to decrease the memory requirements for running the registration scripts provided in the Github repository for this project. Subject images were also rigidly aligned with the template prior to this study to support easy and rapid visualization of subject anatomy in standard anatomical planes.

To compare intensity-only registration and label-informed intensity registration approaches, subjects were registered to the template image using the **antsRegistration()** and **labelImageRegistration()** functions in the ANTsR package [9,13,61,62]. The **labelImageRegistration()** function enables anatomically-aware registration by integrating anatomical label maps and optional intensity image pairs into the optimization process. In this study, both label maps and intensity image pairs were used. The registration proceeds in a flexible multi-stage framework: (i) initialization using either a user-supplied transform or a linear alignment (*e.g*., rigid, affine) based on the centers of mass of corresponding label maps; (ii) deformable registration using intensity-based similarity metrics (typically via the SyN algorithm), initialized by the output of the first stage; and (iii) optional refinement using a label-based similarity metric. All label images remain fixed during optimization and serve as biologically meaningful anchors that guide deformation, and individual labels may be differentially weighted to emphasize structures of interest. In the intensity-only pipeline, the linear initialization was derived from direct registration of the intensity images; in contrast, the label-informed pipeline initialized alignment using center-of-mass alignment of the organ label maps. Both approaches then proceeded through deformable registration using intensity image similarity. All registrations excluded non-subject voxels using manually segmented whole-subject masks. Importantly, the same functionality is available in the Python-based ANTsPy library via the **label_image_registration()** function. Registration scripts and associated image data are available at https://github.com/raroston/labelImageRegistration_Comparison.

### Label overlap

To compare the results of intensity-only and label-informed registration approaches, the Dice statistics were calculated between registration-derived labels (output labels) and the manual (input) labels generated by an expert morphologist. Registration-derived labels were generated by transforming atlas labels into subject space using the inverse transforms from both intensity-only and label-informed image registration. The Dice statistic were calculated using the **labelOverlapMeasures()** function in ANTsR [13,61–63].

### Visualization

To visualize the accuracy of image alignment by intensity-only registration and label-informed intensity registration, each subject (yellow) was transformed into the coordinate system of the template (*i.e.,* template space) and overlaid onto the template (cyan) with the alpha (opacity) channel of both images set to 0.5 in 3D Slicer. Accurate registration should produce a perfect overlap of the transformed subject over the template, producing a green color. Anatomical regions with a poor overlap of the transformed subject over the template appear as yellow or cyan.

### Organ volume analysis

Physical volumes of labels were calculated using the **labelStats()** function of ANTsR and Mann-Whitney U tests were performed using base R functions [9,13,61–63]. The Mann-Whitney U tests compared organ volumes between *Gli2*^*-/-*^ and *Gli2*^*+/+*^ subjects for each organ; organ volumes were calculated from manual segmentations generated by an expert morphologist and serve as a ground-truth reference against which the TBM results are evaluated.

### Tensor Based Morphometry (TBM)

In tensor-based morphometry (TBM), Jacobian determinants are compared between groups to identify statistically significantly different local shape and size differences [24]. The Jacobian determinant is a scalar value calculated from a deformation field (*i.e.,* transform) that reflects local volume changes associated with the deformation. Jacobian values greater than zero indicate local volumetric expansion and values less than zero indicate local volumetric contraction. In this study, the forward transforms deform wildtype and knockout subjects into the space of the template image. Thus, the Jacobian values indicate local volume expansion and contraction involved in transforming subjects into the template space. By statistically comparing Jacobian values on a per-voxel basis between genotypes, local shape and size differences can be identified in any part of the embryo (*e.g.,* both within organs and in the spaces between organs). TBM results are shown as a heatmap; voxels displayed in the heatmap are statistically significantly different between wildtype and knockout subjects and voxel colors indicate the value of the Jacobian determinant.

Jacobian determinants were calculated on a per-voxel basis from the forward transforms generated from each registration approach using the **CreateJacobianDeterminantImage()** function of ANTsR [9]. For each registration approach, a voxel-wise linear regression (voxel ~ genotype) was performed to compare the Jacobian determinants from wildtype and knockout subjects. The p-values were adjusted for multiple comparisons using false discovery rate (FDR < 0.05) by using the relevant functions in the ANTsR and base R libraries [9,13,61–63]. Heatmaps of significantly different Jacobian determinant values were visualized in 3D Slicer [59,60].

### Principal Component Analysis (PCA)

Principal component analysis (PCA) of the deformation fields (*i.e.,* transforms) output by a registration is one way to visualize which subjects are morphometrically similar. Each point on a PC plot represents the deformation that superimposes the subject and template image or the deformation that superimposes the template image onto the subject. Thus, subjects that plot closer together on an axis are more morphometrically similar on that axis.

PCA was conducted on combined inverse transforms using the **multichannelPCA()** function in ANTsR [9,61]. Because subjects were registered to the template image, the forward transforms transform subjects into template space and the inverse transforms transform the template image into subject space. PCA can be performed on either set of transforms with equivalent results; by using the inverse transforms in PCA, we are able to visualize morphological changes along a PC axis by transforming the template image with the eigenvector. To create visually discernable transforms associated with each PC axis, eigenvectors were scaled by −500 (negative PC extrema) and 500 (positive PC extrema) and used to transform the template image into subject space. Multiple scale factors were tested and ±500 was found to produce a discernable transformation of the template image without introducing unrealistic distortions or clipping of the image.

## Results

### Overt phenotypes in *Gli2*^*-/-*^ subjects

*Gli2*^*-/-*^ (knockout) subjects show severe scoliosis with sharp bends and twists in the spinal cord and misshapen or missing vertebrae, causing the torso to appear longitudinally compressed and relatively wide (Fig 1, S1 Fig). Compared to *Gli2*^*+/+*^ (wildtype) subjects, the cranial base of the knockout subjects has a less acute angle with the overall body axis.

### Image alignment and registration quality

#### Dice overlap between manual and registration-based labels.

To compare the results of intensity-only and label-informed registration approaches, Dice statistics were calculated between registration-derived organ labels and manual organ labels that were segmented by an expert in morphology for each subject (Fig 2). For wildtype subjects, both registration approaches resulted in relatively accurate image alignment; nearly every organ in every subject had a Dice statistic greater than 0.75 (Fig 2). The mean of the organ-level Dice statistics from intensity-only registration was 0.93 ± 0.04 and from label-informed registration was 0.95 ± 0.03 (Fig 2).

But, for knockout subjects, intensity-only registration resulted in poor alignment with the template image; Dice statistics for individual organs and subjects ranged from 0 to 0.97 and, for each organ, showed substantially lower means and greater standard deviations than intensity-only registration of wildtypes (Fig 2). The mean of the organ-level Dice statistics from intensity-only registration of knockout subjects was 0.58 ± 0.31. Label-informed registration greatly improved the alignment, as the mean of the organ-level Dice statistics increased to 0.84 ± 0.19 in label-informed registration (Fig 2). The amount of improvement varied by organ, but, with the exception of the rectum and right thymic rudiment, the Dice statistic for each organ and subject increased to greater than 0.75 as a result of the label-informed registration approach.

#### Overlay of knockouts transformed into template space over template.

To further visualize the quality of registration output, subjects were transformed into template space and overlaid over (*i.e.,* superimposed over) the template (Fig 3). To qualitatively assess the transformations generated by intensity-only registration and label-informed intensity registration, each subject (yellow) was transformed into the coordinate system of the template (*i.e.,* template space) and overlaid onto the template (cyan) with the alpha (opacity) channel of both images set to 0.5 in 3D Slicer. As such, anatomical regions with a poor overlap of the transformed subject over the template appear as yellow or cyan, and areas of overlap appear green.

In wildtype subjects, intensity-only and label-intensity registration both produced similar overlays; regardless of registration approach, the superimposing transformed subjects onto the template shows similar alignment of organs (Fig 3B,3D). However, in knockout subjects, intensity-only registration did not result in accurate alignment with the template; most organs in knockout subjects transformed into template space only partially overlapped the corresponding organ in the template (Fig 3A). In contrast, label-informed intensity registration resulted in a much closer alignment of knockout subjects with the template, especially in regions with labels (Fig 3C).

Many registration-based morphometric analyses are performed using only the deformable registration output; often, the initial linear transform primarily reflects differences in overall scale (*i.e.,* size), while deformable transforms capture the remaining differences in shape after scaling. To compare the linear transforms calculated by the two registration approaches, subjects were transformed using the forward linear transform and superimposed on the template. The superimposed images showed that the initial linear transforms calculated in the two registration approaches produced different scaling results in knockout and wildtype subjects in this study (Fig 3E–3H). In intensity-only registration, knockout subjects were scaled to approximate the template image (Fig 3E) and then deformable transforms locally expanded and shrank the subject image (Fig 3A). In contrast, in the label-intensity registration, many of the knockout subjects fit around the template (Fig 3G) and then the deformable transforms primarily shrank parts of the image to align with the template (Fig 3G). In wildtype subjects, both registration approaches produced similar linear transformations (Fig 3F,3H). Consequently, due to the difference in scaling, subsequent analyses to compare the registration approaches used combined linear and deformable transforms.

### Tensor-based morphometry

#### Label-informed TBM yields greater statistical power.

**Fig 4:**
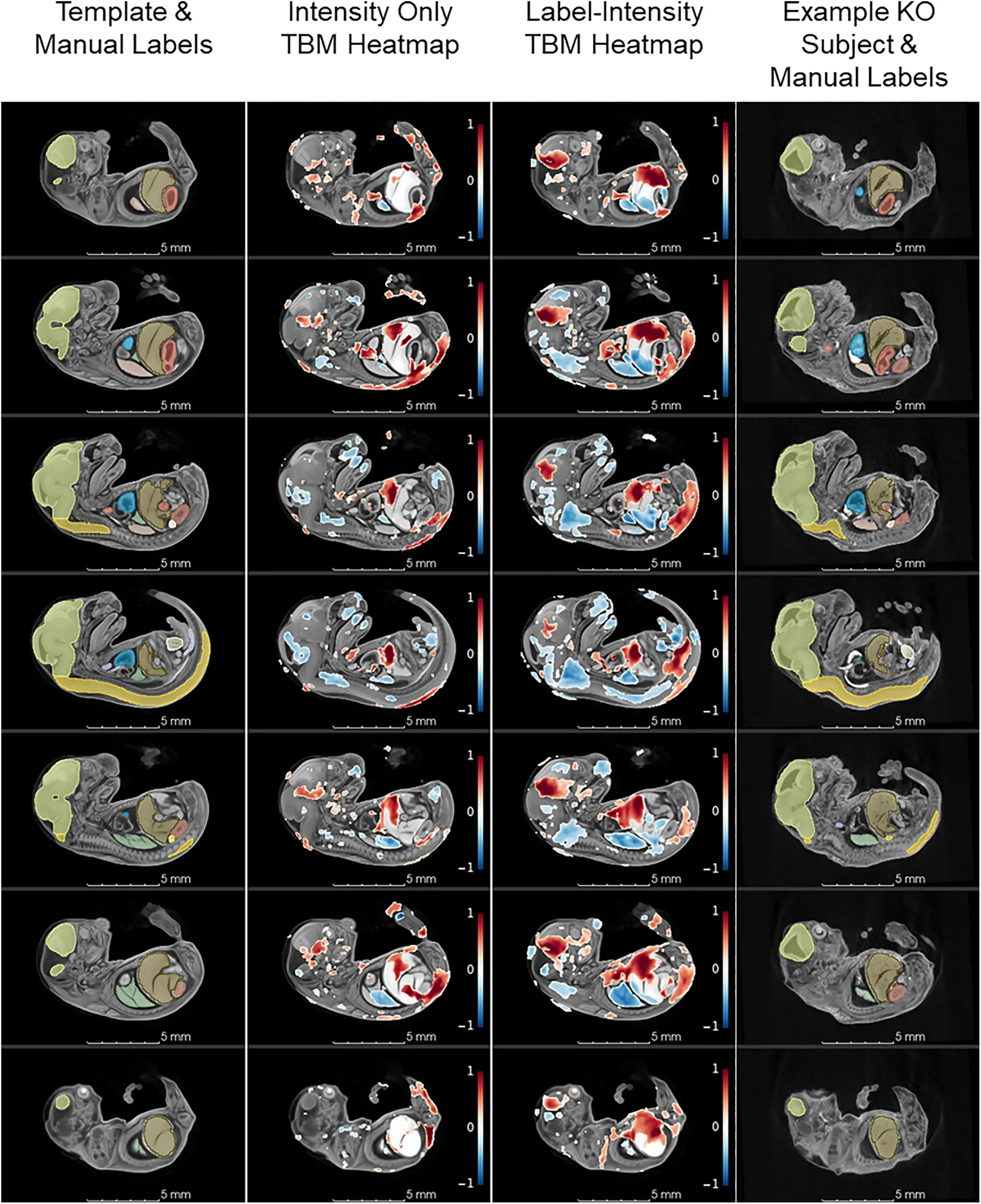
TBM heatmaps shown in serial sagittal slices in the template image. (left) Organ labels used in label-informed image registration; (middle left) TBM result based on intensity-only registration; (middle right) TBM result based on label-informed image registration; (right) example knockout subject (Scan_0105) showing morphological features typical of the *Gli2*^*-/-*^ knockout embryos. Label-informed TBM showed a 50% increase in statistically significant (FDR-adjusted p < 0.05) Jacobian determinants compared to intensity-only TBM and a 45% increase in the range of Jacobian values. Label-informed TBM shows larger regions and magnitudes of volume reduction in the face, neck, lungs, liver, spine, bladder, and vesicorectal septum and volume expansion in the brain, thoracic cavity, liver, spine, and retroperitoneum. For comparative purposes, the heatmaps only show Jacobian values between −1 and 1. For organ names, see Fig 1.

Tensor-based morphometry (TBM) compares the Jacobian determinants calculated from transformations to identify statistically significant voxel-wise differences in local volume (Ashburner & Friston 2000). Significantly different Jacobian values between knockout and wildtype subjects (FDR-adjusted p < 0.05) are shown as a heatmap overlaid over the template image (Fig 4). The magnitudes in the heatmap represent the magnitudes of the Jacobian determinant; negative Jacobian values indicate local volume reduction in knockout subjects relative to wildtypes and positive Jacobian values indicate local volume expansion in knockout subjects compared to wildtypes.

TBM using label-informed image registration identified 53% more significantly different Jacobian values than TBM using intensity-only registration. Of the 1,255,976 voxels in the template image, tensor-based morphometry (TBM) using label-informed image registration identified 159,129 voxels (12.67%) as having significantly different Jacobian values while TBM using intensity-only registration only identified 103,707 (8.26%) (Fig 4).

The range of Jacobian magnitudes also increased in label-informed image registration compared to intensity-only registration. Jacobian values calculated from intensity-only registration transforms ranged from −0.99 to 1.94 whereas Jacobian values calculated from label-informed intensity registration transforms was −0.96 to 2.34, marking a 45% increase in range and greater sensitivity to local volume differences.

#### TBM results are consistent with label-derived volume measurements.

**Fig 5:**
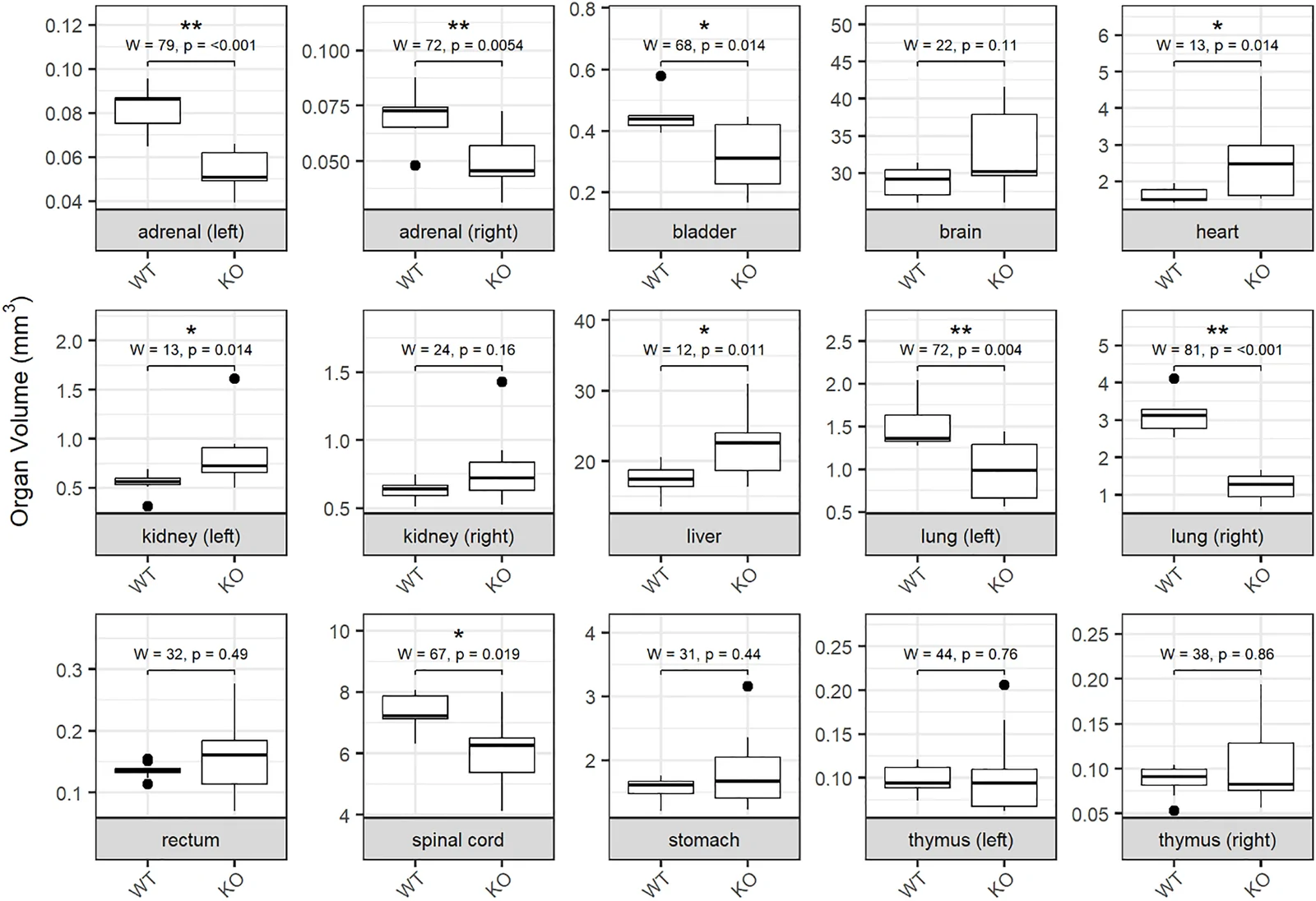
Organ volumes measured from labels manually segmented by a morphology expert and compared using the Mann-Whitney test. The W statistic of the Mann-Whitney test and p value are reported in all panels. Stars indicate different levels of statistical significance (** indicates p < 0.001; * indicates p < 0.05; no stars indicate p > 0.05).

TBM results with both registration methods are grossly consistent with organ volume measurements calculated from manually-segmented organ labels (the ground-truth) (Fig 5). Organs that are smaller in volume in knockout subjects, such as the bladder and lungs, show negative Jacobian values in both registration approaches (Figs 4 and 5). Likewise, organs that are larger in knockout subjects, such as the heart ventricles and liver, show positive Jacobian values in both registration approaches (Figs 4 and 5).

#### Label-informed TBM reflects overt gross morphological differences.

**Fig 6:**
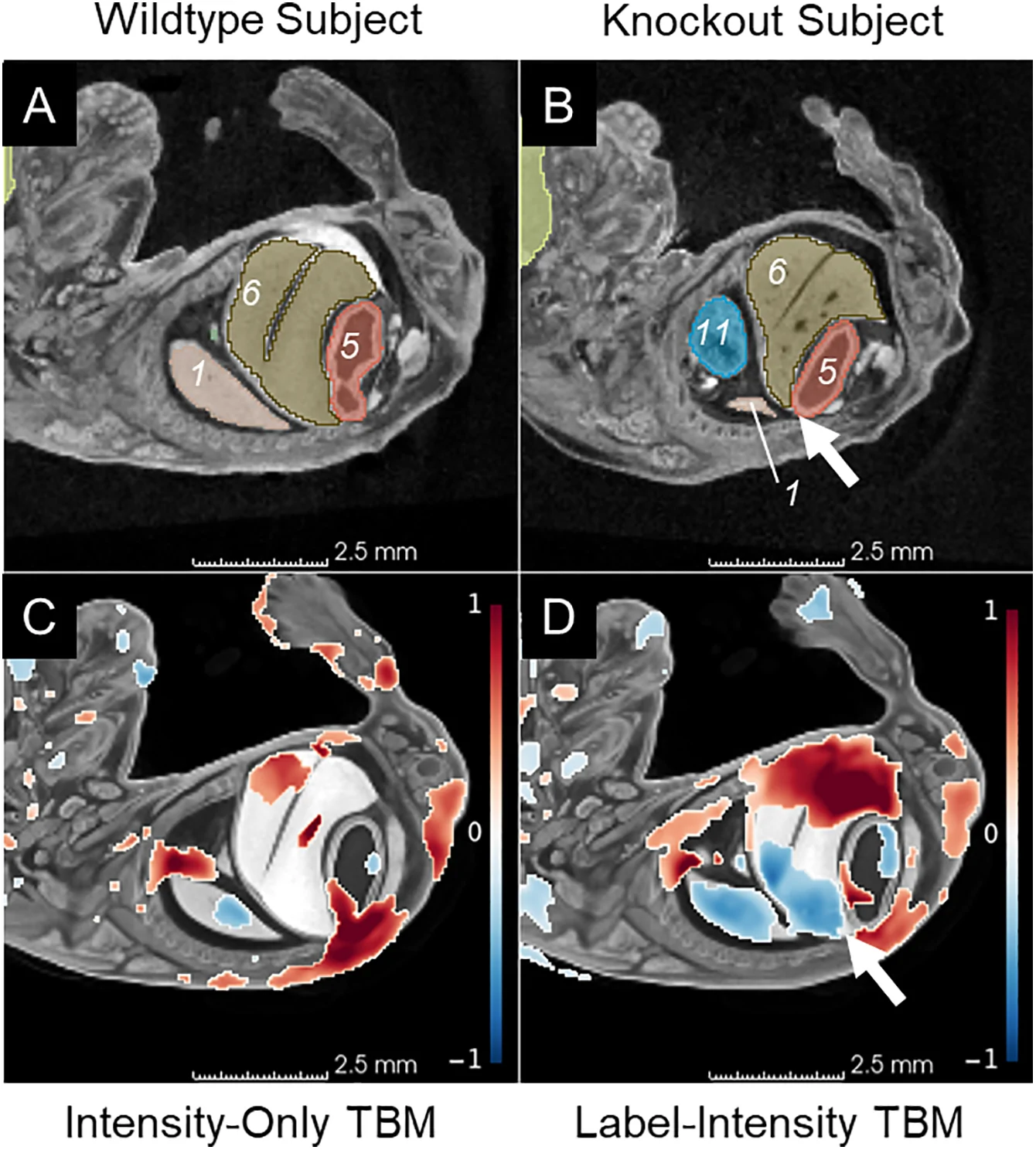
Anterodorsal displacement of the stomach in a parasagittal slice. (A) A representative wildtype subject (Scan_0093) showing normal positions of the stomach and liver; (B) a representative knockout subject (Scan_0110) showing anterior displacement of the stomach and ventral displacement of the liver associated with longitudinal compression of the torso; (C) intensity-only TBM heatmap (D) label-informed TBM heatmap overlaid onto the template image. White arrows indicate the anterior displacement of the stomach and ventral expansion of the liver in the knockout and the corresponding region in the TBM heatmap. red = local volume expansion, positive Jacobians values, blue = local volume reduction in knockouts relative to wildtype subjects. Numbers indicate organ label: *1 left lung, 5 stomach, 6 liver, 11 heart ventricles.*

Within several organs that did not have significantly different label volumes (FDR-adjusted p > 0.05), label-informed TBM captured a combination of local volume increases and decreases, indicating differences in volumetric organ shape, which were not captured by intensity-only TBM (Figs 4 and 5). For example, multiple knockout subjects show anterodorsal displacement of the stomach with ventral displacement of the liver (Fig 6); in at least two subjects, this displacement is also associated in some subjects with a diaphragmatic hernia (not shown). Volumes calculated from organ labels showed no significant difference in stomach volumes and a significant increase in liver volume in knockout subjects compared to wildtypes (Fig 5). Intensity-only and label-informed TBM both showed that, compared to wildtypes, the knockout subjects had a volume expansion in a dorsal portion and a small volume reduction in an anterior portion of the stomach, and localized volume expansion in the ventral portion of the liver. However, label-intensity TBM also showed a volume reduction in the dorsal portion of the liver near the diaphragm, which was not captured in intensity-only TBM. The volume reduction captured by label-intensity reflects the ventral shift of the liver and anteroposterior compression of the dorsal liver, which is readily observable morphology in the knockout embryos (Fig 6B).

**Fig 7:**
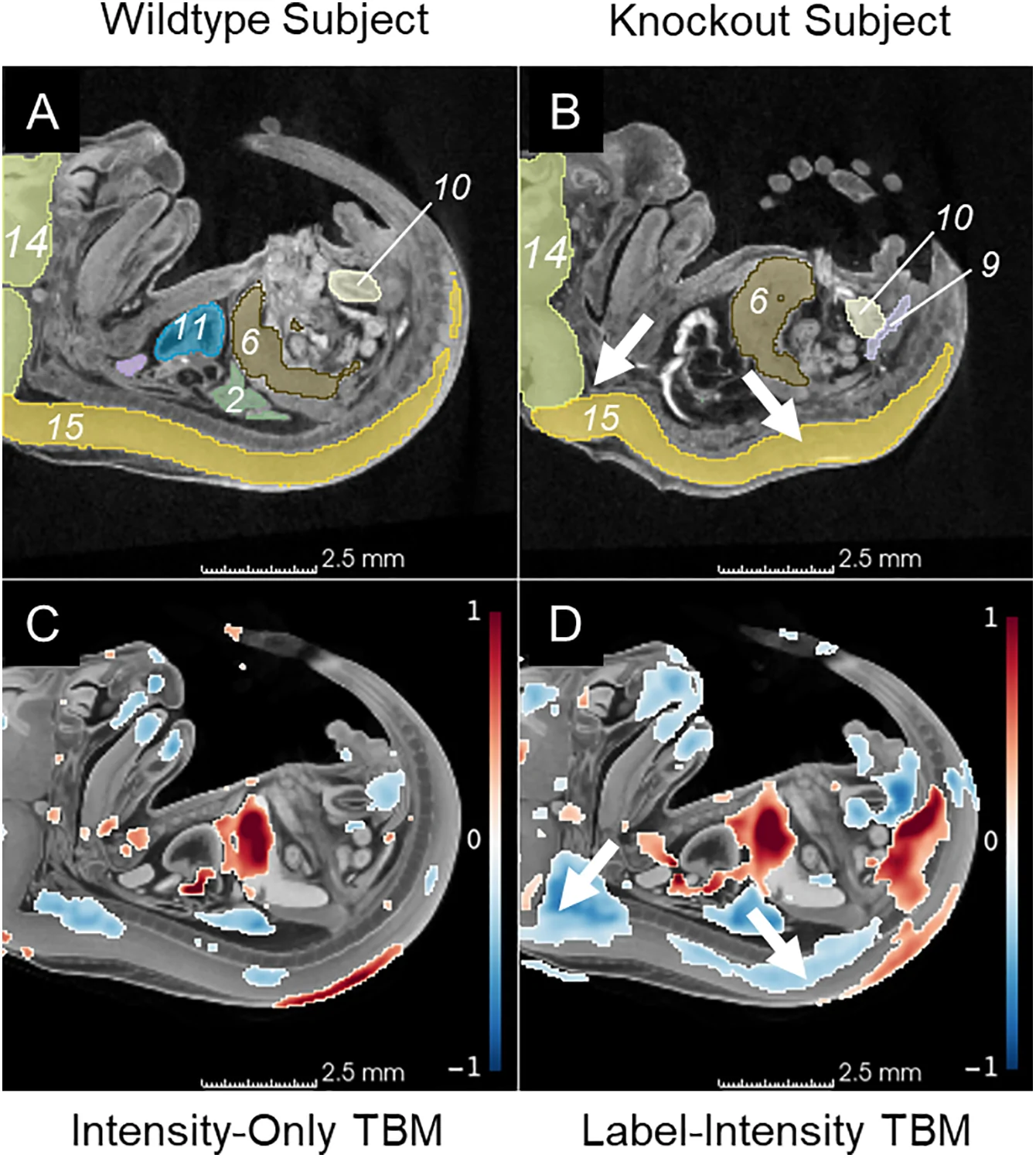
Scoliosis in a sagittal slice. (A) A representative wildtype subject (Scan_0093) showing normal positions of the stomach and liver; (B) a representative knockout subject (Scan_0105) showing severe bends and twists in the spinal cord and vertebral column; (C) intensity-only TBM heatmap and (D) label-informed TBM heatmap overlaid onto the template image. White arrows indicate the sharp bends in the knockout subject and corresponding negative (blue) Jacobian values in the TBM heatmap. red = local volume expansion, positive Jacobians values, blue = local volume reduction in knockouts relative to wildtype subjects. Numbers indicate organ label: *2 right lung, 6 liver, 9 rectum, 10 bladder, 11 heart ventricles, 14 brain, 15 spinal cord.*

A similar phenomenon is also observed in the spinal cord. The segment-based organ volumes show that knockout subjects have a smaller spinal cord than wildtypes (FDR-adjusted p < 0.05). However, the label volume alone gives no indication of the severe scoliosis observed in knockout subjects. Intensity-only TBM shows small regions of ventral volume reduction in the cervical and lumbar regions of the spinal cord with a small region of volume expansion in the posterodorsal portion of the lumbar region closer to the rump. Label-intensity TBM heatmap shows much larger statistically significantly different regions as well as larger Jacobian magnitudes than intensity-only registration; and, the regions of volume difference more closely correspond with sharp bends in the spinal cord observed in knockout subjects (Fig 7B).

**Fig 8:**
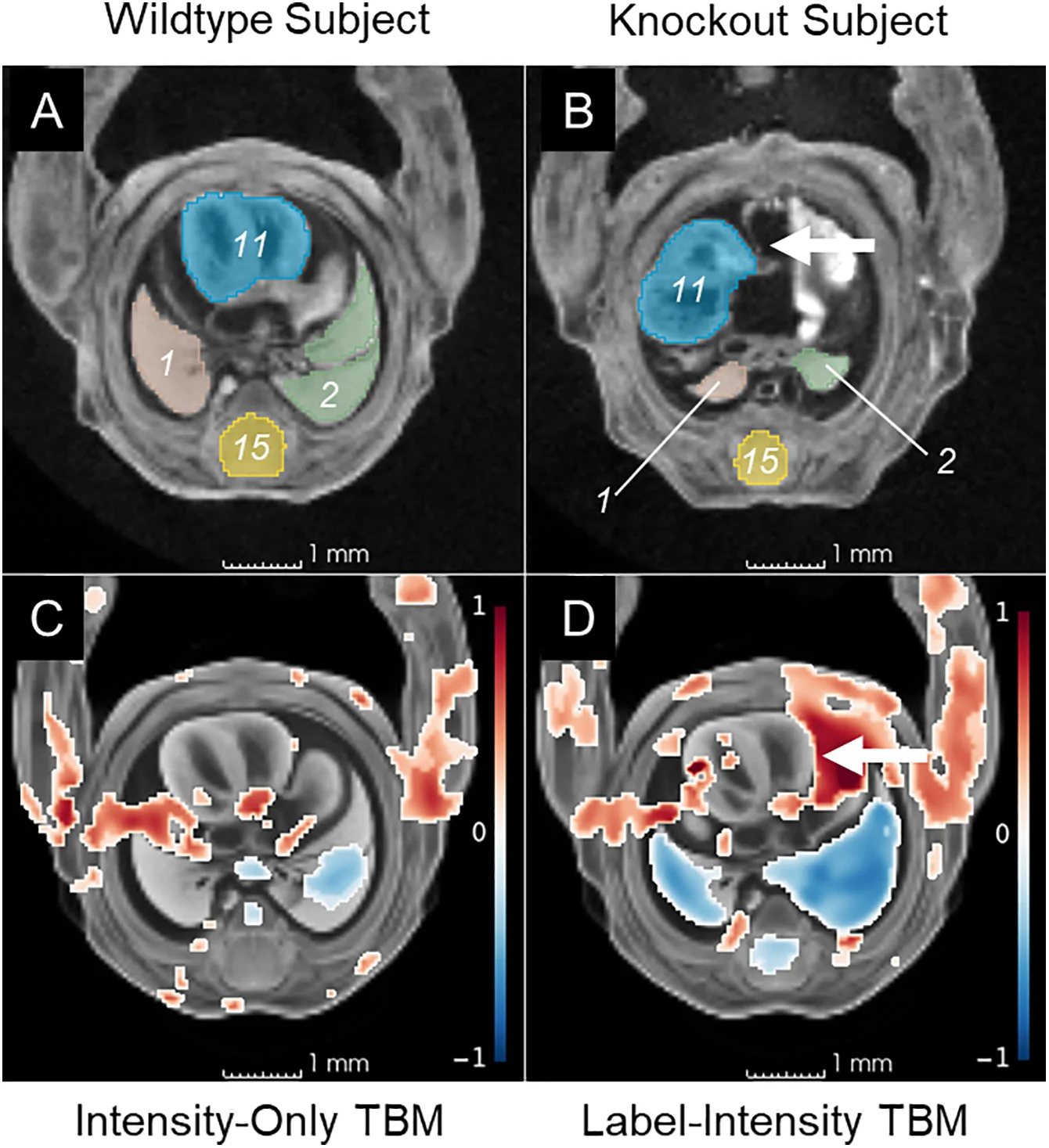
Transverse slices showing displacement of the heart ventricles toward the left side of the thoracic cavity. (A) A representative wildtype subject (Scan_0093) showing the typical position of the heart ventricles within the thoracic cavity; (B) a representative knockout subject (Scan_0110) showing leftward displacement of the heart ventricles (C) Intensity-only TBM heatmap and (D) label-informed TBM heatmap overlaid onto the template image. White arrows indicate the leftward shift of the heart in the knockout subject and corresponding positive Jacobian values indicating local volume expansion in the TBM heatmap. red = local volume expansion, positive Jacobians values, blue = local volume reduction in knockouts relative to wildtype subjects. Numbers indicate organ label: *1 left lung, 2 right lung, 11 heart ventricles, 15 spinal cord.*

In body regions outside of the organ labels, label-intensity TBM also showed increased power and sensitivity compared to intensity-only registration. One example is a large region of volume expansion to the right of the heart ventricles (Fig 8). Intensity-only registration indicated only a small amount of volume expansion in this region, which includes both the right atrium of the heart and the right pleural space. Coronal slices of the thoracic cavity in knockout subjects clearly show that in the majority of subjects, the heart is displaced toward the left of the thoracic cavity, corresponding with the volume expansion observed in label-informed TBM (Fig 8). Knockout subjects also show a qualitative reduction in the volume of both the left and right lungs, and only a single lobe in the right lung, which normally has four lobes (Fig 8); this radical volume reduction is clearly shown in label-informed TBM, but intensity-only TBM only shows a small reduction in the centers of the right lung (Fig 7B) and left lung (Fig 4, not seen in the plane of Fig 8).

### Label-informed PCA captures genotype-associated phenotypes

Principal component analysis (PCA) is a dimensionality reduction technique that allows visualization of morphospace and how individual subjects occupy morphospace. PCA on the combined inverse transforms provides a way to visualize how the template image is transformed to align with individual subject images. Combined transforms from a single subject are represented by a single point in the plot; points that plot closer together on PC axes are expected to indicate that the subjects are more morphologically similar.

PCA on transforms generated from intensity-only registration does not recover known phenotypic differences between knockout and wildtype subjects. Even though there are obvious and highly penetrant phenotypic differences in knockout and wildtype subjects, such as severe scoliosis (see Fig 1, S1 Fig, Fig 7), PCA on the inverse transforms output by intensity-only registration did not separate wildtype and knockout subjects on PC1 or PC2, or any other PC. In other words, intensity-only PCA showed wildtype and knockout subjects overlapping in morphospace, even though knockout subjects have known overt morphological differences from wildtypes.

**Fig 9:**
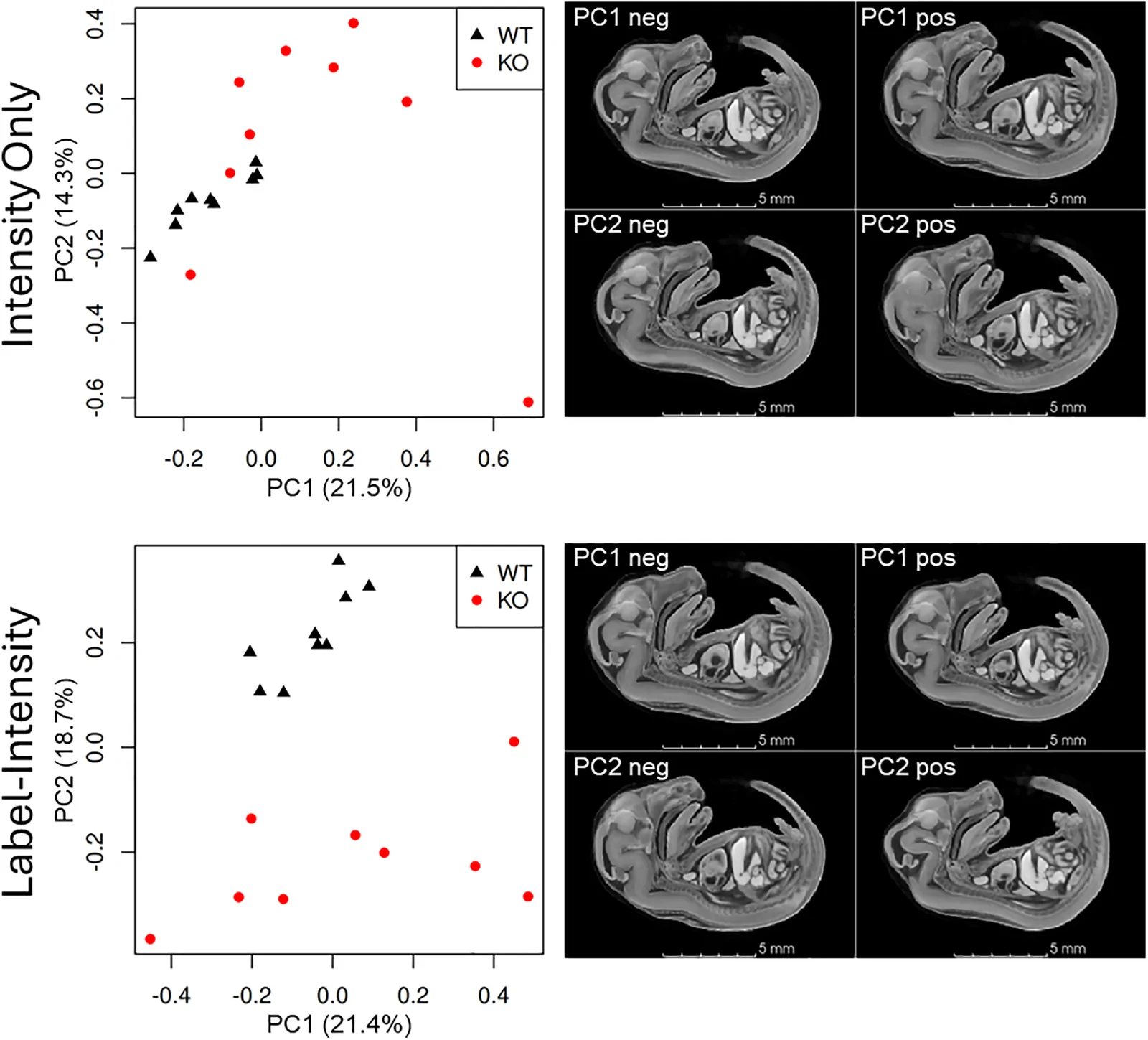
PC plots for combined inverse transforms from intensity-only registration (top) and label-intensity registration (bottom). Wildtype and knockout phenotypes separate along PC2 using transforms from label-informed registration, but do not separate on any PC in intensity-only registration. Transformation of the template image along PC2 shows overall morphology--including severe scoliosis, enlarged heart, and reduced bladder size--consistent with observable phenotypic differences.

In contrast, PCA on the combined inverse transforms from label-informed intensity registration did separate wildtype and knockout subjects on PC2 (Fig 9). Transformation of the template image along PC2 shows that morphological differences along PC2 are consistent with observable, overt phenotypes in knockout subjects; these include: differences in the subjects’ spinal curvature (*i.e.,* scoliosis), an increase in heart ventricle volume, differences in head shape and orientation, and a reduction in the space between the bladder and rectum (associated with a vesicorectal fistula). PC1 primarily reflects overall subject size, as indicated by the increase and decrease in embryo volume in the PC1-associated transformations of the template image (Fig 9).

## Discussion

Label-informed image registration is a recently developed function in the ANTsX ecosystem that extends the capabilities of deformable image registration by incorporating anatomical knowledge via segmentation labels into the alignment process. Conceptually, this approach bridges the longstanding divide between atlas-based segmentation workflows and voxel-based morphometrics by allowing labeled structures to actively inform the registration, rather than serve merely as potential downstream outputs. Historically, intensity-based registration emerged as a powerful method for subtle morphometric analysis in neuroimaging, but difficulties can arise with large morphological variation, particularly in developmental and comparative studies where anatomy may be missing, shifted, or grossly deformed. Label-informed registration provides a solution by offering anatomically anchored initialization and refinement, thus enabling meaningful correspondence across highly divergent morphologies. This functionality is publicly available as part of the well-vetted, open-source ANTsR and ANTsPy libraries, which are widely used in biomedical imaging research and maintained with extensive community and peer-reviewed support. Importantly, the underlying nonlinear optimization is driven by the Symmetric Normalization (SyN) algorithm, the high-performing registration framework at the core of ANTsX, which has repeatedly demonstrated top-tier performance in comparative evaluations. By building on a foundation of robust, validated methods and extending them with anatomically-aware capabilities, this tool supports more accurate and interpretable morphometric analyses in cases where traditional registration techniques are prone to failure.

### Improved image registration and morphometrics

Overt phenotypic differences in E15.5 *Gli2* knockout embryos have caused intensity-only registration to the wildtype template image to fail in large regions of the subject or for the entire image. *Gli2* knockout subjects differ in overall geometry from wildtype subjects due to severe scoliosis and large displacements of internal organs (Fig 1). These overt differences have caused intensity-only registration of knockout subject images to the wildtype template image to fail in large regions of the subject or for the entire image. Label-informed image registration provided more accurate registration of these knockout subjects to a wildtype template image based on qualitative visual assessment of overlaid images. More accurate registration increased the power of downstream morphometric results using both tensor-based morphometry (TBM) and principal component analysis (PCA).

Because both wildtype and knockout subjects had better alignment with the template image, the calculated transformations (registration output) better reflect observable, qualitative morphological differences (Figs 5–8). TBM using both registration approaches showed local volume differences that were grossly consistent with measured label volumes; but, TBM using label-informed intensity registration captured additional local volume differences not captured by intensity-only TBM that reflect observable differences in organ shape.

Likewise, PCA on intensity-only registration transforms did not show any genotype-associated phenotypic differences—wildtype and knockout subjects did not separate on any PC axis—despite readily apparent overt phenotypic differences. Because intensity-only registration failed in several knockout subjects, the transforms used in this PCA do not reflect real morphological differences and the results are unreliable. In contrast, PCA on label-informed intensity registration transforms separated genotype-associated phenotypes on PC2, while PC1 primarily captured overall subject size. Thus, as expected, PCA on label-informed intensity registration transforms showed genotype is one of the main sources of morphological variation.

These results suggest that the integration of label-informed image registration into phenotypic analysis pipelines can expand the range of phenotypes, and therefore genotypes, that can be studied in reverse genetic screens. This registration approach can consequently increase the applicability of existing analytical pipelines to a wider range of genes, phenotypes, and research questions [31,34,50–52].

### Segmentation as a registration input

Manual image segmentation is a time-intensive process requiring substantial anatomical expertise [58]. Just a few years ago, it would have been considered unfeasible to generate labels to use as an input for image registration for a larger number of images. Rather, labels have been one of the many potential derived data generated from finding correspondences in images through registration (*e.g.*, [64]). In atlas-based (registration-based) segmentation, a single template, or atlas, is expertly segmented, subjects are registered to the template image, and then the correspondences obtained through image registration are used to transform the atlas labels to match individual subjects [31,34,64].

Numerous semi-automated tools and deep learning models now exist to make segmentation relatively quick and easy (*e.g*., [12,58,65–69]). For example, the Segment Editor module in 3D Slicer and the SlicerMorph extension provide semi-automated tools to speed up image segmentation [12,59,60]. Promptable deep learning-based frameworks like nnInteractive harness the benefits of deep learning while allowing users more control of segmentation [67,68]. Several pre-trained models also provide fully-automated segmentation of multiple organs [58,65,69]. It is also now easier to train task-specific segmentation models from a small amount of training data through aggressive data augmentation [28,70]. Because segmentations can now be generated relatively easily and independently of image registration, labels can now be used as an input for registration.

Many current anatomy-aware registration approaches are task-specific; that is, they perform well in the specific context for which they were designed for (*e.g.*, [36–38]). The label-informed image registration function in the ANTsX ecosystem offers a general-purpose solution for anatomy-aware registration for a wide array of morphological variation. Because the labels are generated completely separately from the registration, they can be generated with any segmentation approach–manually, using semi-automated segmentation tools, or using task-specific deep learning models. This flexibility and customizability, allows users more options for defining regional correspondences to guide the registration.

### Limitations of label-informed intensity registration

Label-informed intensity registration allows the registration of more diverse (or, geometrically dissimilar) morphologies and this greater diversity can add additional considerations when comparing individuals and populations. For example, there are many ways to choose a baseline for scaling subjects with different overall shapes depending on the morphological question [5]. Differences in overall shape between subjects or populations can also affect the scaling produced by linear transforms, and therefore the ability to use the linear transforms as a proxy for scale. In the present study, severe scoliosis causes *Gli2* knockout subjects to be relatively anteroposteriorly compressed compared to wildtype counterparts and to have a different angle between the head and torso (Fig 1, S1 Fig). As a result, the linear transforms calculated using label-informed intensity registration increased the overall scale of knockout subjects to fit the subject around the template image, rather than approximate knockout subjects to the template (Fig 3). Then, the deformable transforms primarily shrank the image to fit the template. While this particular outcome is likely specific to the *Gli2* knockout phenotype and the specific labels selected for this study, it indicates that scaling from label-informed image registration may differ from other registration methods and between different morphologies, and that scaling may not be consistent between intensity image and label datasets.

While most registration methods are optimized to produce consistent results, label-informed image registration results can be biased by label choice, the distribution of labels across the image, and the relative weighting of each label. Selecting labels concentrated in only one region of an image could skew the registration toward that region and cause poor registration of other regions. Selected labels may also cause large or unrealistic transformations that are not relevant or informative. Additionally, labels must be present in all of the images being registered, which means that corresponding structures must be present in all the images. This limits, for example, the registration of subjects at later developmental stages to earlier developmental stages in which certain structures may not yet have developed. Further research is needed to examine how label choices, distributions, and weights may affect registration outcomes.

### Potential applications of label-informed image registration

#### Label-guided atlas-based segmentation.

Depending on the research question, deep learning-based segmentation may not be an adequate replacement for atlas-based segmentation. While it has improved significantly, deep learning-based segmentation has several inherent limitations. Supervised training can require large amounts of well-annotated training data, which may be difficult to generate [71]. Additionally, inference is often poor for novel data that was not included in the training dataset [72]. And, unlike atlas-based segmentation which results in smooth transformations of atlas labels, deep learning-based segmentation can introduce small islands and other noise that can be difficult to detect. In many cases, it is likely that registration accuracy can be greatly improved with the introduction of only a small number of initial labels in a label-informed registration approach. Then, the more accurate transforms could be used to transfer additional labels from an atlas to subjects using an atlas-based segmentation approach.

#### Developmental and comparative morphometrics.

Landmark-based geometric morphometric approaches have become commonplace in ontogenetic and inter-species comparative studies (*e.g.,* [8,19,22,49,73,74]). But, landmarks often provide relatively sparse sampling of a volumetric (voxel) image and capture only a small portion of the overall shape variation [7,16]. Surface-based morphometrics can also provide detailed shape analysis, but do not capture the internal structure of anatomical features (*e.g.*, [18]). Previously, registration-based morphometrics has been restricted to subjects with a high degree of morphological similarity and has been difficult to apply in comparative contexts. Label-informed image registration offers a way to conduct volumetric morphometric studies on a broader range of phenotypic diversity including different species and different developmental stages in which homologous regions can be identified.

## Supporting information

S1 Fig Gross morphological differences in knockout embryos compared to wildtype embryos. The spinal cord and vertebral columns of *Gli2*^*-/-*^ (knockout) embryos have severe bends and twists (*i.e.,* scoliosis), which alters the overall body shape and topology of internal organs.
(PNG)
