# Supplementary material for: Anatomy-aware, label-informed approach improves image registration for challenging datasets
Source: PLoS One. 2026 Sep 8;21(9):e0357448. doi: 10.1371/journal.pone.0357448 (PMC13552761; doi:10.1371/journal.pone.0357448)
Supplement: Supplementary file 1 [file pone.0357448.s002.pdf]

Roston et al. Anatomy-aware, label-informed approach improves image registration for challenging datasets

Reviewer Comments

Roston et al. introduce a label-informed image registration method implemented within the ANTsX ecosystem, designed to improve registration quality for anatomically complex datasets. The authors benchmark their approach using DiceCT images of Gli2-/- knockout and wildtype mouse embryos at developmental stage E15.5. They demonstrate that incorporating segmentation labels prior to registration improves the spatial correspondence to the selected template compared to intensity-only approaches and enhances downstream volumetric morphometric analyses. The basic concept is valuable and the topic is timely, particularly for developmental and evolutionary studies where registration quality strongly influences morphological inferences. The paper is promising, but requires substantial revisions concerning clarity, structure, quantitative support and methodological transparency. My detailed comments follow.

Abstract

The Abstract contains a typographic error ("intensityonly") but this appears to be a submission-system error as it is not present in the manuscript page. The phrase "registration knockout mouse embryos" is syntactically incorrect, as I believe an "of" is missing. The abstract also feels a little over descriptive in terms of the general properties of ANTsC rather than specifically summarising the study's findings. Sentences explaining how labels can be generated are too generic, the abstract should instead state which label were used in the present study and how they were obtained. Instead of just using the term knockout mice, I think the Gli2 gene should be explicitly mentioned in the Abstract rather than being first mentioned in the introduction. The term "downstream statistical analysis" is also too vague, the abstract should specify which analyses (e.g. volumetric comparisons, TBM, PCA).

Overall, the abstract needs to be more focused on study-specific results and less on generic capabilities, so the readers can quickly understand what has been done and why it matters.

Introduction

I think there needs to be some work on the clarity and wording. The phrase “despite overt differences in morphology” would be better expressed as “even when there are overt differences in morphology”. The original wording suggestions a contradiction, which is not necessarily the case, morphological comparisons can be done in morphologies with little disparity. The term “qualitative phenotypic differences” also feels inappropriate in this context; “phenotypic differences” is sufficient and avoids confusion with categorical traits.

The early paragraphs are somewhat disjointed. The movement from traditional measurements to modern geometric techniques is important but could be more tightly linked to the specific topic of volumetric morphometrics and registration-based pipelines, which is the paper’s actual focus. The discussion of the morphometric history presently includes an unnecessary detour to D’Arcy Thompson. While historically relevant, the connection to paper is less apparent. A more useful framing would be to (1) define linear morphometrics (i.e. callipers), (2) introduce geometric morphometrics, (3) mention algorithmic approaches such as LDDMM, ICP and polynomial-based descriptors (e.g. Euler characteristics), (4) state their downfalls, and then (5) link the need for volumetric and voxel-based approach of VBM and TBM. All these topics should be framed explicitly in relation to why a label-informed registration method is needed, thus aligning with the subsequent methodological developments in the paper.

The discussion around imaging modalities also is hard to follow. The current text refers to imaging advances without specifying modalities. Since the data involve micro-CT, you should introduce 3D imaging modalities (e.g. MRI, micro-CT) and connect these modalities to the challenge of generating accurate image registrations and then clarify how these fit into the ANTsX ecosystem.

The flow of the following paragraphs could also be improved. The discussion of VBM and TBM should include concrete examples with citations, ideally involving mouse development datasets (e.g. studies using 3D images for VBM comparisons) to demonstrate the current errors with intensity-only registration-based methods. The points made in paragraphs 4 and 5 contain related arguments and should be merged and reorganised to avoid redundancy. The discussion of limitations is informative but overly long. Condensing this section and restructuring it to lead directly into the introduction of the new anatomy-aware approach would greatly improve clarity.

Paragraphs 7-11 are also too long and interrupt the narrative. They contain useful information, but they need to be reduced and integrated more succinctly. Given that ANTsX is already well documented, the introduction should provide only the essential information and move quickly towards what the study novelly contributes.

Methods

The methods are generally clear and readable, but several key areas require clarification or expansion. The manuscript should explicitly describe how antsRegistration differs from labelImageRegistration. Is the difference solely the use of segmentation masks, or are there additional implementation differences? A high-level explanation of the label-informed cost function would also improve accessibility.

The authors use t-tests, but it is unclear whether normality was tested. Given that earlier results suggest poor performance of intensity-only registration, the underlying data distributions may deviate from normality. Normality tests should be reported, or a justification for parametric tests should be provided. Similar concerns apply to the Jacobian determinant data in the TBM analysis.

The TBM section needs more details. This includes (a) a formal definition of the Jacobian determinant, (b) an equation for its computation within this context and (c) a brief interpretation of what local expansion/ contraction signifies biologically. This information

would make the section more accessible to readers not already familiar with TBM frameworks.

The PCA and inverse transforms section also requires some improvement. There needs to be a clearer explanation of what the inverse transformations represent, again preferably with an equation to back this up. There needs to be justification of why the range -500 to 500 was used in the scaling; did you test any alternatives?

Results

In the first paragraph of the "Label-informed image registration improves Gli2-/- registration" in the Results, the text should include reference to Figure 3 to clarify what is being discussed.

Currently, the results include lots of qualitative comparisons of the data rather than quantitative comparisons. I feel these are needed to help substantiate lots of your claims. For example, to compare the improved correspondence using the label-based registration techniques, you could use the following techniques to quantify the overlaps: Dice similarity coefficient, Intersection over Union (IoU), Surface distance metrics and quantitative measures of scaling differences between registration modes. Without more quantitative backing, claims of improvement remain subjective and biased.

Figures 4-7 while conceptually useful, are difficult to interpret in their current form. These issues specifically are related to (a) insufficient clarity of heatmaps, (b) ambiguous alignment with corresponding input images and (c) limited explanation in captions. To improve these I would suggest: (1) placing the original labelled images in the first row, followed by the heatmaps, (2) use consistent colour palettes to label segments in these figures aligned with those in Figure 1 to further highlight which organs are being compared, which means adding segmentation labels to assist interpretation and (3) combining related figures of 5-7 to improve the interpretation. For Figure 4 you should explicitly state that the heatmaps compares the wildtype vs knockout differences. In this figure and following 5-7 I would

change the plan; I would consider adding two columns showing the manual labels for the wildtype and knockout samples (with colour labels for organs) and then the two heatmap differences in the predictions done using the intensity-only and label-intensity approaches. There just requires increased guidance on what is being compared in Figure 4.

For the voxel number differences (i.e. 159,129 voxels) I would also assign percentages to these in terms of relation to the overall number of voxels.

Table 2 also conveys limited information as it currently only included manually generated data. To make table more useful could you: (1) include the intensity-only registration predicted volumes, (2) then indicate which correlations are statistically significant by highlighting them in bold and (3) providing more details on the differences in the predictions of the volumes between methods. I think this would help clarify the issue of volume predictions on the results. You could also consider doing a linear regression plot comparing the volumes generated across each of the methods and perform a Pearson or Spearman's rank correlation comparison.

The comparison of the PCA space could also be substantially stronger with additional statistical analyses. You could for one fit a Gaussian mixture model (e.g. via mclust in R with n = 2) to evaluate how clustering would be assigned under a null and then compare this classification vs those obtained with the manually assigned values in the two different PCA spaces across the two methods. You should also evaluate the agreement between the intensity-only and label-guided methods numerically to show how much they differ. To do this you could use a combination of the Procrustean randomisation tests (PROTEST) to compare the matrix shapes, pairwise Euclidean distance comparisons between a selected point and the correlations between the PC scores. These would convert a subjective visual assessment into a robust statistical support.

Discussion

Currently, the discussion has promising elements but lacks cohesion and sufficient referencing. There is need for additional references to substantiate claims. For instance, claims about the limitations about intensity-only registration and its issues with applying to developmental anatomy require references. There also needs to be references related to foundational VBM/TBM papers in its use. There should also be references about the ANTs/ANTsX methodology and any prior work of each of these techniques comparing knockout and wildtype developmental morphologies. This would help to support the issues with current methods.

Many points on the discussion rely on qualitative interpretation (e.g. “closer match”, “greater accuracy”). These statements must be substantiated quantitatively through the suggested metrics (Dice, IoU, PCA distances etc.).

The discussion around segmentation inputs is important, but the authors do not frame this within the context of their paper adequately. There are practical costs (i.e. time and accuracy) of using different methods to obtain segmentation labels, but their effects on the results of the TBM is not shown. It would be useful to provide a time comparison between the intensity and label-based registration techniques to see how much time manual labelling adds to the time. You could also test the feasibility of using semi-automated or automated segmentation methods of generating the label data required for the label-based method. A comparison of the manual vs automated label maps on the downstream results would be useful to backup the points made in this discussion section.

The structure of the discussion could also be improved. It would benefit from stronger linkage between the (a) methodological issue of previous methods, (b) the results of the study being used to demonstrate how they help overcome these issues and their limitations in addressing these issues, and (c) what are the broader implications of this? For instance, there needs to be greater discussion about the implications of being able to compare knockout and wildtypes through developmental stages. Additionally, the manuscript is

missing a conclusion section. A short summary outlining the key findings and future directions would greatly improve readability and synthesis.

Other Points

Figure 2 could be moved to the Supplementary material to make room for clearer comparative figures. Comparison of PC3/PC4 results could also be added to a supplemental.

The data is easily accessible via the provided GitHub link. Here the code for performing the analysis can also be found. I have not run the code due to the size of the outputs, but I am happy with the accessibility.

Overall, the study presents a promising and potentially valuable advance in registration-based morphometrics. The use of label-informed registration is conceptually strong and appears to offer meaningful improvements, particularly for anatomically challenging datasets such as malformed embryos. However, the manuscript requires substantial revisions to improve clarity, strengthen narrative flow, and most importantly provide quantitative evidence supporting claims of superiority over intensity-only registration. With additional analyses and improved cohesion, this paper has the potential to make a significant contribution to volumetric morphometric methodology.
