# Supplementary material for: Anatomy-aware, label-informed approach improves image registration for challenging datasets
Source: PLoS One. 2026 Sep 8;21(9):e0357448. doi: 10.1371/journal.pone.0357448 (PMC13552761; doi:10.1371/journal.pone.0357448)
Supplement: Supplementary file 2 [file pone.0357448.s004.docx]

We thank the reviewer for their thoughtful comments and recommendations. We made substantial revisions to the manuscript to improve clarity, strengthen the narrative flow, and provide quantitative evidence supporting the claims. The revisions we made are detailed below.

Abstract

- We fixed typographic errors detailed by the reviewer
- The goal of the paper is to provide an example of applying a new registration method that has been added to the ANTs ecosystem and discuss its generic capabilities and applicability. Characterization of the Gli2 knockout phenotype was not the main goal of the manuscript, rather Gli2 knockout mice serve as an example of overt morphological differences that have impeded registration by other methods. The generalizations in the abstract referenced by the reviewer were intentional choices by the authors, aligned with the goal of the manuscript. To clarify our intended goal, wording was revised throughout the abstract and introduction.
  - Reviewer’s comments: “*“The abstract also feels a little over descriptive in terms of the general properties of ANTsC rather than specifically summarising the study’s findings. Sentences explaining how labels can be generated are too generic, the abstract should instead state which label were used in the present study and how they were obtained. Instead of just using the term knockout mice, I think the Gli2 gene should be explicitly mentioned in the Abstract rather than being first mentioned in the introduction. The term “downstream statistical analysis” is also too vague, the abstract should specify which analyses (e.g. volumetric comparisons, TBM, PCA). Overall, the abstract needs to be more focused on study-specific results and less on generic capabilities, so the readers can quickly understand what has been done and why it matters.“*

Introduction

- We Incorporated wording suggested by the reviewer:
  - “…even when there are overt differences in morphology”
  - We changed “qualitative phenotypic differences” to “overt morphological differences” throughout the manuscript, and revised the manuscript for more consistent wording of “overt morphological differences”
    - Reviewer’s statement: *“I think there needs to be some work on the clarity and wording. The phrase “despite overt differences in morphology” would be better expressed as “even when there are overt differences in morphology”. The original wording suggestions a contradiction, which is not necessarily the case, morphological comparisons can be done in morphologies with little disparity. The term “qualitative phenotypic differences” also feels inappropriate in this context; “phenotypic differences” is sufficient and avoids confusion with categorical traits.”*
  - Revised discussion of the evolution of morphometric techniques in accordance with reviewer suggestions:
    - Reviewer’s statements: *“The early paragraphs are somewhat disjointed. The movement from traditional measurements to modern geometric techniques is important but could be more tightly linked to the specific topic of volumetric morphometrics and registration-based pipelines, which is the paper’s actual focus. The discussion of the morphometric history presently includes an unnecessary detour to D’Arcy Thompson. While historically relevant, the connection to paper is less apparent. A more useful framing would be to (1) define linear morphometrics (i.e. callipers), (2) introduce geometric morphometrics, (3) mention algorithmic approaches such as LDDMM, ICP and polynomial-based descriptors (e.g. Euler characteristics), (4) state their downfalls, and then (5) link the need for volumetric and voxel-based approach of VBM and TBM. All these topics should be framed explicitly in relation to why a label-informed registration method is needed, thus aligning with the subsequent methodological developments in the paper.”*
- Reorganized the introduction of image registration and knockout mouse phenotyping, including adding sub-titles and streamlining the narrative:
  - We added a new sub-title “Image Registration and revised and reorganized the text to:
    - Succinctly describe the “traditional” approach of intensity-only registration
    - Defined the concept of “anatomy-aware” approaches and briefly describe existing “anatomy-aware” methods
    - Explained how our anatomy-aware approach is different than existing methods and introduce it as new functionality in the ANTsX ecoystem
    - Reviewer’s statement: *“The discussion around imaging modalities also is hard to follow. The current text refers to imaging advances without specifying modalities. Since the data involve micro-CT, you should introduce 3D imaging modalities (e.g. MRI, micro-CT) and connect these modalities to the challenge of generating accurate image registrations and then clarify how these fit into the ANTsX ecosystem.”*
    - Reviewer’s statement: *“The points made in paragraphs 4 and 5 contain related arguments and should be merged and reorganised to avoid redundancy. The discussion of limitations is informative but overly long. Condensing this section and restructuring it to lead directly into the introduction of the new anatomy-aware approach would greatly improve clarity. Paragraphs 7-11 are also too long and interrupt the narrative. They contain useful information, but they need to be reduced and integrated more succinctly. Given that ANTsX is already well documented, the introduction should provide only the essential information and move quickly towards what the study novelly contributes.”*
  - We added text to better explain the role of image registration in mouse phenotyping and our goal of using mouse phenotyping as an example for the use of label-informed image registration. We added text to explain the need for high-throughput, automated phenotyping pipelines for reverse genetic screens, the key role for image registration in proposed analytical pipelines, the use of TBM in these pipelines, and the limitations of “traditional” intensity-only registration for this application. TBM is that it is a common technique described in detail elsewhere and was used in this paper because it is commonly used in the phenotype analysis pipelines for knockout mice on which we based this manuscript.
    - Reviewer comment: *“The flow of the following paragraphs could also be improved. The discussion of VBM and TBM should include concrete examples with citations, ideally involving mouse development datasets (e.g. studies using 3D images for VBM comparisons) to demonstrate the current errors with intensity-only registration-based methods.”*

Methods

- We added a new section explaining the labelImageRegistration method in greater detail
  - Reviewer comment: *“The methods are generally clear and readable, but several key areas require clarification or expansion. The manuscript should explicitly describe how antsRegistration differs from labelImageRegistration. Is the difference solely the use of segmentation masks, or are there additional implementation differences? A high-level explanation of the label-informed cost function would also improve accessibility.”*
- We changed the statistical test for organ volume data to non-parametric Mann-Whitney U tests instead of t-tests. To better visualize the distributions of organ volume measurements, we also added a box and whiskers plot of organ volumes. We added clarification in the text that our main goal for including volumetric measurements is as a ground-truth to which to compare TBM results. For the TBM analysis, we utilized a voxel-wise linear regression under the framework of the General Linear Model (GLM), which is the standard, well-established approach for voxel- and tensor-based morphometry (Ashburner & Friston, 2000). While the GLM does carry an assumption of normally distributed errors, prior methodological work has demonstrated that voxel-wise group comparisons in balanced or nearly balanced designs are highly robust to violations of normality (Salmond et al., 2002). Therefore, we have retained the GLM/linear regression approach for the TBM analysis. However, we realized that we inadvertently referred to this as a 't-test' in the Figure 4 caption and the Results text, which caused understandable confusion. We have corrected these typos throughout the manuscript to accurately reflect the use of linear regression.
  - Reviewer comment: *“The authors use t-tests, but it is unclear whether normality was tested. Given that earlier results suggest poor performance of intensity-only registration, the underlying data distributions may deviate from normality. Normality tests should be reported, or a justification for parametric tests should be provided. Similar concerns apply to the Jacobian determinant data in the TBM analysis.”*
- We added text to better explain the Jacobian determinant in this context, the steps of TBM, and the biological interpretation of TBM results
  - Reviewer comment: *“The TBM section needs more details. This includes (a) a formal definition of the Jacobian determinant, (b) an equation for its computation within this context and (c) a brief interpretation of what local expansion/ contraction signifies biologically. This information would make the section more accessible to readers not already familiar with TBM frameworks.”*
- We added text to better explain the application and interpretation of PCA in this context, the decision to use inverse transforms in this analysis, and the choice of the scaling range -500 to 500. This scaling does not alter the direction of the eigenvectors. It simply increases or decreases the magnitude of the transformations to make the morphological changes along the PC axes visually discernible without introducing unrealistic distortions or clipping.
- We added a section on Dice calculations
- We revised section on visualizations for clarity

Results

- Text was changed throughout the Results section to clarify the finding that that morphometrics resulting from intensity-only registration did not reflect real, observable morphological differences and that only label-informed registration was able to align knockout images with the template such that the morphometrics reflect observable morphological differences:
  - This finding was clarified by the incorporation of Dice scores in Figure 2, text regarding overlaid images, and the text explaining Figures 4, 5-8
  - We thank the reviewer for suggesting methods to more deeply interrogate the PCA spaces. While statistical methods such as Gaussian mixture models and PROTEST are excellent approaches for comparing morphospaces in typical evolutionary or developmental studies, we have opted not to perform these statistical comparisons here because doing so would misrepresent the nature of our data. The fundamental basis of the PCA in this context is the output of the deformable registration. As demonstrated by multiple lines of evidence, including the Dice statistics and visual overlays, the intensity-only registration fundamentally failed to accurately align the knockout subjects. Consequently, the PCA space derived from the intensity-only registration does not represent a valid biological morphospace; rather, it represents registration error. Applying robust statistical comparisons between the two PCA spaces would falsely imply that both registration methods produced valid morphometric results that can be meaningfully benchmarked against one another, which our data clearly show is not the case.
    - Reviewer comment: *“The comparison of the PCA space could also be substantially stronger with additional statistical analyses. You could for one fit a Gaussian mixture model (e.g. via mclust in R with n = 2) to evaluate how clustering would be assigned under a null and then compare this classification vs those obtained with the manually assigned values in the two different PCA spaces across the two methods. You should also evaluate the agreement between the intensity-only and label-guided methods numerically to show how much they differ. To do this you could use a combination of the Procrustean randomisation tests (PROTEST) to compare the matrix shapes, pairwise Euclidean distance comparisons between a selected point and the correlations between the PC scores. These would convert a subjective visual assessment into a robust statistical support.”*
- Overall structure of the Results section and sub-titles were revised for clarity and revised figure references
  - Reviewer’s comment: *“In the first paragraph of the “Label-informed image registration improves Gli2-/- registration” in the Results, the text should include reference to Figure 3 to clarify what is being discussed.”*
- We added a section on Dice coefficients (a new plot and text) as a quantitative measure of registration accuracy
  - Reviewer’s comment: *“Currently, the results include lots of qualitative comparisons of the data rather than quantitative comparisons. I feel these are needed to help substantiate lots of your claims. For example, to compare the improved correspondence using the label-based registration techniques, you could use the following techniques to quantify the overlaps: Dice similarity coefficient, Intersection over Union (IoU), Surface distance metrics and quantitative measures of scaling differences between registration modes. Without more quantitative backing, claims of improvement remain subjective and biased.”*
- Table 2 was removed and a new figure was added showing box and whisker plots for organ volumes to allow better visualization of the mean and variance of organ volumes. Text was added and revised to clarify that these measurements were included as a ground-truth for the TBM results. Because the Dice statistic for intensity-only registration was so low, we do not think it is useful to show predicted volumes (which would be inaccurate). Rather, we added text to clarify that the organ volumes calculated from manual segmentations provide a ground-truth to which to compare the TBM results (*i.e.,* the TBM results are consistent with measured organ volume differences)
  - Reviewer comments: *“Table 2 also conveys limited information as it currently only included manually generated data. To make table more useful could you: (1) include the intensity-only registration predicted volumes, (2) then indicate which correlations are statistically significant by highlighting them in bold and (3) providing more details on the differences in the predictions of the volumes between methods. I think this would help clarify the issue of volume predictions on the results. You could also consider doing a linear regression plot comparing the volumes generated across each of the methods and perform a Pearson or Spearman’s rank correlation comparison.”*
- Figures 4-7 (now Figures 4, 6-8) were revised based on the reviewer’s recommendations. Figure 4 now includes an additional column showing an example knockout subject and Figures 6-8 (formerly 5-7) were revised so that example wildtype and knockout subjects are in the top row. Organ labels matching Figure 1 were added to Figures 6-8 for additional clarity.
  - Reviewer’s comment: *“Figures 4-7 while conceptually useful, are difficult to interpret in their current form. These issues specifically are related to (a) insufficient clarity of heatmaps, (b) ambiguous alignment with corresponding input images and (c) limited explanation in captions. To improve these I would suggest: (1) placing the original labelled images in the first row, followed by the heatmaps, (2) use consistent colour palettes to label segments in these figures aligned with those in Figure 1 to further highlight which organs are being compared, which means adding segmentation labels to assist interpretation and (3) combining related figures of 5-7 to improve the interpretation. For Figure 4 you should explicitly state that the heatmaps compares the wildtype vs knockout differences. In this figure and following 5-7 I would change the plan; I would consider adding two columns showing the manual labels for the wildtype and knockout samples (with colour labels for organs) and then the two heatmap differences in the predictions done using the intensity-only and label-intensity approaches. There just requires increased guidance on what is being compared in Figure 4.”*
- Percentages were added to the voxel number differences for TBM
  - Reviewer comment: *“For the voxel number differences (i.e. 159,129 voxels) I would also assign percentages to these in terms of relation to the overall number of voxels.”*

Discussion

- Clarifications requested by the reviewer were primarily added to the Introduction rather than the Discussion to provide better clarity for the rationale and goals of this manuscript:
  - The references suggested by the reviewer regarding developmental anatomy and VBM/TBM were incorporated into the introduction of the manuscript to better clarify the context for our manuscript and the methodological issues of previous methods were addressed in the Introduction
    - Reviewer comment: *“Currently, the discussion has promising elements but lacks cohesion and sufficient referencing. There is need for additional references to substantiate claims. For instance, claims about the limitations about intensity-only registration and its issues with applying to developmental anatomy require references. There also needs to be references related to foundational VBM/TBM papers in its use. There should also be references about the ANTs/ANTsX methodology and any prior work of each of these techniques comparing knockout and wildtype developmental morphologies. This would help to support the issues with current methods.”*
  - The major advantage of this label-informed registration approach is that segmentations are generated separately from the registration. This is unlike other anatomy-aware methods that often integrate segmentation directly into the registration pipeline. This point was clarified through changes made to the introduction of the manuscript. The authors agree that assessing the influence of different segmentation choices on registration outcomes is an important area for future study, and added text to the Discussion to reflect this.
    - Reviewer comment: *”* *The discussion around segmentation inputs is important, but the authors do not frame this within the context of their paper adequately. There are practical costs (i.e. time and accuracy) of using different methods to obtain segmentation labels, but their effects on the results of the TBM is not shown. It would be useful to provide a time comparison between the intensity and label-based registration techniques to see how much time manual labelling adds to the time. You could also test the feasibility of using semi-automated or automated segmentation methods of generating the label data required for the label-based method. A comparison of the manual vs automated label maps on the downstream results would be useful to backup the points made in this discussion section.”*
  - Multiple sections were added to the introduction clarifying the methodological issues of previous methods (both anatomy-aware methods and knockout mouse phenotyping pipelines) and the results of this study that show that intensity-only registration largely failed in knockout mice but label-informed registration produced a better superimposition of the images. We aimed to clarify the broader implications of this method by clearly defining “overt morphological differences” in the introduction, using that phrase consistently, and explaining how overt morphological differences can affect registration.
    - *“The structure of the discussion could also be improved. It would benefit from stronger linkage between the (a) methodological issue of previous methods, (b) the results of the study being used to demonstrate how they help overcome these issues and their limitations in addressing these issues, and (c) what are the broader implications of this? For instance, there needs to be greater discussion about the implications of being able to compare knockout and wildtypes through developmental stages.*
- Other points:
  - Figure 2 was changed to Supplemental Figure 1
  - As stated in the plot of PCs 1 and 2 show that the morphometrics based on intensity-only registration failed to capture real, observable morphological differences. Additional plots for the other PCs can be generated using the data and scripts available in the Github repository. We have added text to the readme explaining how to request a MorphoCloud instance to run the analyses
    - Reviewer comment: *“Comparison of PC3/PC4 results could also be added to a supplemental. The data is easily accessible via the provided GitHub link. Here the code for performing the analysis can also be found. I have not run the code due to the size of the outputs, but I am happy with the accessibility.”*
