# Supplementary material for: Anatomy-aware, label-informed approach improves image registration for challenging datasets
Source: PLoS One. 2026 Sep 8;21(9):e0357448. doi: 10.1371/journal.pone.0357448 (PMC13552761; doi:10.1371/journal.pone.0357448)
Supplement: Supplementary file 3 [file pone.0357448.s005.docx]

We thank the reviewers for their thoughtful comments and suggestions.

Reviewer #1: The authors have satisfactory addressed the concerns raised in the initial review or have provided adequate justification where changes were not implemented. Prior to publication, however, several minor issues should be addressed to further improve the manuscript:
1. There is an extraneous space at the beginning of the third section of the Image Registration section of the Introduction.

*We fixed a typo of a double space at the beginning of a sentence, but did not notice any other extraneous space.*

2. In the first sentence of the fourth paragraph of the Automated Phenotyping of Genetic Knockouts, the word “have” in “we have selected a genetic” is not needed and can be removed.

*Removed the word “have”*

3. The final paragraph of the Introduction reads more like a conclusion. Given the substantial length of the introduction, its inclusion may not be necessary. If retained, it should be substantially reduced. Alternatively, a better option may be to revise this and incorporate it into a dedicated concluding section, which is currently lacking. This will help to provide a more holistic view of the overall study.

*We thank the reviewer for this comment. For clarity, the authors prefer to provide a preview of the results and conclusions at the end of the introduction. Doing so helps orient the reader as they read the Methods, Results, Discussion. Many readers read scientific papers out of the order in which they were written, so we believe providing guide posts (even if slightly repetitive) in different sections helps with clarity.*

4. The results presented in Table 2 could be integrated into Figure 5. Specifically, the W and p-values could be included within each organ-specific panel, thereby eliminating the need for Table 2. Additionally, the Figure 5 caption is currently too brief and should be expanded. At the bare minimum this should be to define the abbreviation of “NS” and to provide a clearer summary of the results shown.

*The information from Table 2 was added to Figure 5 and Table 2 was removed.*

5. In Figure 1, please verify that all organ labels are clearly visible. For example, labels “6” and “7” are not readily apparent, and the label “1” on the coronal slice of the second panel is difficult to discern.

*Edited Figure 1 to add labels 7 and 8 and moved label 1 to the left of the embryo*

6. Figure 2 is informative but could be improved. If the boxplot format is retained, consider adding a line indicating the mean value across all organs within each panel. However, a more effective alternative may be to present combined regression plots comparing values obtained from the two registration methods (i.e. Intensity-only vs Label-Informed) for both WT and KO datasets. Here the confidence intervals and labels for each individual organ can still be added into the plot.

*A dotted line indicating the mean Dice statistic was added to each panel.*

7. In Figure 3, the yellow color label appears somewhat orange; a more clearly distinguishable color may help improve clarity. Additionally, ensure that the text labels match the corresponding colors used in the figure. To improve readability, consider adding headings above each of the four panel groups to indicate the associated transformations (Combined forward transforms vs linear transforms only).
*Added labels for the associated transformations. Lightened the yellow text to increase contrast against the black background. The figures are true yellow and cyan, but appear as different shades in part due to reduced opacity against the black background.*

8. Figures 6-8 may be excessive for the main text and could be moved to the supplementary material. Many of the patterns they illustrate cane be observed in Figure 4 upon closer inspection.

*The TBM results are the same in Figure 4 and in Figures 6-8, but Figures 6-8 are meant to highlight how the TBM results correspond with actual morphological differences. The purpose of these figures is to make the main text more accessible to readers who may not be as familiar with all of the anatomical terms we use by providing a visual representation and to allow readers with more background in anatomy to independently assess the anatomy shown in the images rather than relying solely on our descriptions. We therefore think these figures are integral to the manuscript and prefer not to move them to the supplementary material.*

Reviewer #2: Authors developed a registration method by incorporating parcellated label maps into the cost function. Registration is mostly performed using image intensities. In this application, authors proposed to use expert guided segmentations of organs such as brain, spinal cord, liver, and heart from Micro-CT scans of wild type and Gli2 knockout embryos. Authors showed that their proposed approach (combination of label maps and image intensities), compared to intensity based registration, substantially improved registration by showing dice overlap statistics and qualitative examples. In addition, authors showed that accurate derivation of warps to template image provides better quantification of morphological differences between wild type and Gli2 knockout embryos. Provided heat maps derived from voxel based morphometry and plots of principal component analysis are used to demonstrate qualitative and quantitative differences between wild type and Gli2 knockout embryos. Authors generated a template image by averaging 35 wildtype micro-CT scans from 8 inbred and outbred knockout mouse lines. Over 250 embryos collected and scanned for an ongoing reverse genetic screen. Gli2 was selected for this particular study. I have listed my suggestions below:

-Paper is well organized but needs some modifications and clarifications.

-Some references cited several times throughout the manuscript, such as 9, and 32. Some of these repeated citations are unnecessary, I suggest these should be reviewed for necessity. In addition, I see references to books or workshop proceedings, if there are specific parts in this book or proceedings that would be more relevant for the reader to check in case of a need.

“phenotype discovery described by Wong et al. (2014), Dickinson et al. (2016), and
Horner et al. (2021) (32,35,51).” Here, Author/Year and Number references are not in order.

*This is because of the citation management software. We will ensure that the orders match in the publication.*

I think authors should emphasize that their contribution is to develop this labelImageRegistration function as an additional functionality in Ants package. While reading the manuscript I thought this is already available in the package and someone else developed these functions, I also needed to check the website to notice that this is not coming with default installation.

*The second author of this manuscript, Nick Tustison, is one of the original developers of ANTs. The additional functionality of label-image registration was developed by Dr. Tustinson and has already been integrated into ANTsR and ANTsPy (e.g.,* [*https://github.com/ANTsX/ANTsPy/pull/743*](https://github.com/ANTsX/ANTsPy/pull/743)*). This manuscript is the first presentation and demonstration of the method, its utility, and its application to a biological problem in the literature. Label-image registration functionality is automatically included with more recent versions of ANTsR and ANTsPy. To clarify these points, we revised the text at the beginning of the Methods section to emphasize that this is new functionality. There was already sentence in the intro that states that, but we now restate it in the Methods for clarity.*

In the methods section, the E_total equation is given, but w_i is not described in the same sentence, it is not clear how these weights (w_i and w_intensity) are calculated or initialized.
*We added text to clarify this*

In the Embryo Collection and Micro-CT section: It is specified that these subjects were a subset of over 250 embryos, that would be good if authors can explain why we don’t read a certain number here. How many of these embryos were used in the experiments? This is not clear.

*“Over 250 embryos” was used because collection and scanning was ongoing, so the precise number was changing. We have updated the number to reflect the current total number of scans.*

In the Registration section: “Subject images were also rigidly aligned with the template prior to this study to support easy and rapid visualization of subject anatomy in standard anatomical planes.” I think this is also a required step prior to nonlinear warp estimation, that would also be mentioned as a support, this is a suggestion.

*Yes, rigid alignment is one of the components of the linear registration we use in this manuscript. The code we provide in the Github repository assumes that subjects are already in an approximately standardized orientation. If someone were to try to adapt our code to another dataset, they may find it doesn’t work well if their subjects initially have very different orientations. So, we included this sentence to clarify that there was a standardization step that was done previously and not included in this study or in the code provided in the Github repository. (The prior rigid registration is obvious if one opens the scan images provided in the repository, but we thought it was useful to state explicitly that it was a prior step because people may not visualize the images when accessing our code.)*

I think there are a lot of subsections in Methods, such as the Organ Volume Analysis section. This section is not clear and I don’t understand why Mann-Whitney U tests mentioned here, what it compares should be mentioned clearly.

*We revised that section as suggested.*

Results section: “Missing and misshapen vertebrae have been previously described in Gli2-/- mouse embryos (56,64) but the effects of these defects on overall geometry of the vertebral column and morphometrics of the embryo have not been previously studied.” I think this sentence shouldn’t appear in the results, and sentences exist in the introduction, if 56 and 64 are not cited in the introduction that would be good to move this into the introduction section.

*This sentence was moved to the Introduction and removed from the results.*

In section “Dice Overlap between manual and registration-based labels”: I think authors may give how the overall mean dice statistics calculated, when I look at Figure 2 and whisker box plots of intensity only registration of knockout subjects I would expect to have a mean value smaller than 0.81, but I’m not sure about this suggestion. Please consider this as a minor note to authors to double check.

*The means we originally presented were calculated from the overall means of the registrations (i.e., calculated from the Dice statistic for all of the labels from a single individual subject, the “All” label in the plot in Figure 5), which we referred to as the “overall Dice statistic.” However, this is inconsistent with how we chose to present the data in Figure 5. So, for clarity and consistency, we now present the mean of the Dice statistics for all of the organ labels, not separated by individual registration.*

In section “Overlay of knockouts transformed into template space over template”: I think an additional comment needed for Figure 3C, where I see yellow region above region iv not warped correctly, is that an insignificant region for the investigation?

*The yellow region above iv is the tail. It was not of interest in this study because the position varies considerably and the variation in positioning is random and not a “structural” difference. We did not have a label in that region, but the alignment of the tail could be improved by including a label there. We have added text to the figure legend to explain this result.*

----I see the figure legends do not match what is being described in this section: in the last paragraph here Figure 3E,F references for knockout subjects, but in the figure F is for wild type; Figure 3G,H referenced for deformable registration, but they are for linear registration. Figure 3E,G referenced for label-intensity but in fact it is not the case I see in the figures.

*Revised the figure references*

In the section “Label-Informed TBM yields greater statistical power”: First paragraph is a repetition, I think that is mentioned in the methods section. I suggest removal of this part.
----“TBM using label-informed image registration identified 50% more significantly different Jacobian values than TBM using intensity-only registration” Is 50% an approximate value? If the exact value is available that would be mentioned here.

Table 2: Caption is not clear, W is not described. Please highlight/mark the p-values which are annotated with single or multiple asterisks in Figure 5. The legends of asterisks are also missing in Figure 5.

*W-statistic*

In section “Label-informed TBM reflects overt gross morphological differences”:
----“this displacement is also associated with a diaphragmatic hernia (Figure 6).” I don’t see any caption information regarding the hernia in Figure 6, that would be great if the hernia is marked in Figure 6.

*Figure 6 does not show one of the embryos with a hernia. The in-text figure references were revised to clarify this.*

----“which is readily observable morphology in the knockout embryos.” I think this sentence needs a citation.

*Added Figure reference*

----“A similar phenomenon is also observed in the spinal cord.” I think this sentence is not clear, so it would be good to refer to the similar observations again.

Figure 8C,D: please mention the description of the background image.

*Slice planes and “overlaid onto the template image” were added to the legends of Figure 6-8 for clarity.*

In section “Label-informed PCA captures genotype-associated phenotypes”:
----First sentence: we don’t need to define PCA again, already mentioned in the methods section.
----“Even though there are obvious and highly penetrant phenotypic differences in knockout and wildtype subjects, such as severe scoliosis (see Figure 1, Suppl. Figure 1),” Why this sentence is not referring to Figure 7, which is about scoliosis.

*Added Figure 7. In an older version of the manuscript the PCA results were presented prior to what are now Figures 6-8 and we overlooked this reference in the revision.*

----“these include: differences in the subjects’ spinal curvature (i.e., scoliosis), an increase in heart ventricle volume, differences in head shape and orientation, and a reduction in the space between the bladder and rectum (associated with a vesicorectal fistula).” I think this sentence should be part of the discussion, it is not good to read this in the results section.
*This sentence is included in the Results section because it specifically describes the morphological differences that result from transforming the template along the PC2 axis: “Transformation of the template image along PC2 shows that* ***morphological differences*** *along PC2…” Following this sentence, we explain the morphological differences observed along the PC1 axis.*

Figure 9: Caption has a reference to Fig 1, this is not good, caption should only describe what is given in the figure.

*Removed the reference.*

Reference 22, check if this is properly cited in the manuscript, I couldn’t locate, but maybe I just missed.

*Reference 22 is listed in the introduction in the range of 20-23:*

*“These methods have been applied to wide variety of study subjects, from plant leaves to vertebrate skulls, and have been applied to a wide variety of biological questions from embryonic development to biological variation to evolution (8,20–23)”*
