# Supplementary material for: Anatomy-aware, label-informed approach improves image registration for challenging datasets
Source: PLoS One. 2026 Sep 8;21(9):e0357448. doi: 10.1371/journal.pone.0357448 (PMC13552761; doi:10.1371/journal.pone.0357448)
Supplement: Peer review history

# Author comment: Author response to Decision Letter 0

**DOI:** 10.1371/journal.pone.0357448.r001

**Transfer Alert**

This paper was transferred from another journal. As a result, its full editorial history (including decision letters, peer reviews and author responses) may not be present.

23 Sep 2025

# Peer review: Decision Letter 0

**DOI:** 10.1371/journal.pone.0357448.r002

**Authors:** Alpen Ortug

5 Jan 2026

Dear Dr. Maga,

Thank you for submitting your manuscript to PLOS ONE. After careful consideration, we feel that it has merit but does not fully meet PLOS ONE’s publication criteria as it currently stands. Therefore, we invite you to submit a revised version of the manuscript that addresses the points raised during the review process.

Please submit your revised manuscript by Feb 19 2026 11:59PM. If you will need more time than this to complete your revisions, please reply to this message or contact the journal office at plosone@plos.org. When you're ready to submit your revision, log on to https://www.editorialmanager.com/pone/ and select the 'Submissions Needing Revision' folder to locate your manuscript file.

- A letter that responds to each point raised by the academic editor and reviewer(s). You should upload this letter as a separate file labeled 'Response to Reviewers'.
- A marked-up copy of your manuscript that highlights changes made to the original version. You should upload this as a separate file labeled 'Revised Manuscript with Track Changes'.
- An unmarked version of your revised paper without tracked changes. You should upload this as a separate file labeled 'Manuscript'.

If you would like to make changes to your financial disclosure, please include your updated statement in your cover letter. Guidelines for resubmitting your figure files are available below the reviewer comments at the end of this letter.

If applicable, we recommend that you deposit your laboratory protocols in protocols.io to enhance the reproducibility of your results. Protocols.io assigns your protocol its own identifier (DOI) so that it can be cited independently in the future. For instructions see: https://journals.plos.org/plosone/s/submission-guidelines#loc-laboratory-protocols. Additionally, PLOS ONE offers an option for publishing peer-reviewed Lab Protocol articles, which describe protocols hosted on protocols.io. Read more information on sharing protocols at https://plos.org/protocols?utm_medium=editorial-email&utm_source=authorletters&utm_campaign=protocols.

We look forward to receiving your revised manuscript.

Kind regards,

Alpen Ortug, PhD

Academic Editor

PLOS One

Journal Requirements:

When submitting your revision, we need you to address these additional requirements.

1. Please ensure that your manuscript meets PLOS ONE's style requirements, including those for file naming. The PLOS ONE style templates can be found at

https://journals.plos.org/plosone/s/file?id=wjVg/PLOSOne_formatting_sample_main_body.pdf and

https://journals.plos.org/plosone/s/file?id=ba62/PLOSOne_formatting_sample_title_authors_affiliations.pdf

2. To comply with PLOS ONE submissions requirements, in your Methods section, please provide additional information regarding the experiments involving animals and ensure you have included details on (1) methods of sacrifice, and (2) efforts to alleviate suffering.

3. Please update your submission to use the PLOS LaTeX template. The template and more information on our requirements for LaTeX submissions can be found at http://journals.plos.org/plosone/s/latex.

4. Thank you for stating the following financial disclosure:

“All funding is to A. Murat Maga from National Institutes of Health (P01HD104435, R03DE031313, S10OD032302)”

Please state what role the funders took in the study. If the funders had no role, please state: "The funders had no role in study design, data collection and analysis, decision to publish, or preparation of the manuscript."

If this statement is not correct you must amend it as needed.

Please include this amended Role of Funder statement in your cover letter; we will change the online submission form on your behalf.

5. Thank you for stating the following in the Acknowledgments Section of your manuscript:

“We thank our colleagues at Seattle Children’s Research Institute and the University of Washington for their contributions to this project: D. Beier, S. Gombart, E. Vincent, and S. Houghtaling for mouse husbandry, genetics, and embryo collection; M. Bell and D. Mao for assistance with specimen preparation and micro-CT scanning; and S. Rolfe, C. Zhang, O. Thomas, and E. Nichols for valuable discussion and feedback on this project. 3D scanning of subjects was conducted at the SCRI MicroCT Imaging Facility (RRID:SCR_024678), using Bruker Skyscan 1272 microCT that was funded by an NIH shared instrument grant (S10OD032302). This work was supported by funding from the National Institutes of Health (NICHD/P01HD104435 and NIDCR/R03DE031313).”

We note that you have provided funding information that is currently declared in your Funding Statement. However, funding information should not appear in the Acknowledgments section or other areas of your manuscript. We will only publish funding information present in the Funding Statement section of the online submission form.

Please remove any funding-related text from the manuscript and let us know how you would like to update your Funding Statement. Currently, your Funding Statement reads as follows:

“All funding is to A. Murat Maga from National Institutes of Health (P01HD104435, R03DE031313, S10OD032302)”

Please include your amended statements within your cover letter; we will change the online submission form on your behalf.

6. If the reviewer comments include a recommendation to cite specific previously published works, please review and evaluate these publications to determine whether they are relevant and should be cited. There is no requirement to cite these works unless the editor has indicated otherwise.

[Note: HTML markup is below. Please do not edit.]

Reviewers' comments:

Reviewer's Responses to Questions

**Comments to the Author**

1. Is the manuscript technically sound, and do the data support the conclusions?

Reviewer #1: Yes

**********

2. Has the statistical analysis been performed appropriately and rigorously?

Reviewer #1: No

**********

3. Have the authors made all data underlying the findings in their manuscript fully available?

The PLOS Data policy

Reviewer #1: Yes

**********

4. Is the manuscript presented in an intelligible fashion and written in standard English?

Reviewer #1: Yes

**********

Reviewer #1: Roston et al. introduce a label-informed image registration method implemented within the ANTsX ecosystem, designed to improve registration quality for anatomically complex datasets. The authors benchmark their approach using DiceCT images of Gli2-/- knockout and wildtype mouse embryos at developmental stage E15.5. They demonstrate that incorporating segmentation labels prior to registration improves the spatial correspondence to the selected template compared to intensity-only approaches and enhances downstream volumetric morphometric analyses. The basic concept is valuable and the topic is timely, particularly for developmental and evolutionary studies where registration quality strongly influences morphological inferences. The paper is promising, but requires substantial revisions concerning clarity, structure, quantitative support and methodological transparency. My detailed comments follow.

The Abstract contains a typographic error (“intensityonly”) but this appears to be a submission-system error as it is not present in the manuscript page. The phrase “registration knockout mouse embryos” is syntactically incorrect, as I believe an “of” is missing. The abstract also feels a little over descriptive in terms of the general properties of ANTsC rather than specifically summarising the study’s findings. Sentences explaining how labels can be generated are too generic, the abstract should instead state which label were used in the present study and how they were obtained. Instead of just using the term knockout mice, I think the Gli2 gene should be explicitly mentioned in the Abstract rather than being first mentioned in the introduction. The term “downstream statistical analysis” is also too vague, the abstract should specify which analyses (e.g. volumetric comparisons, TBM, PCA). Overall, the abstract needs to be more focused on study-specific results and less on generic capabilities, so the readers can quickly understand what has been done and why it matters.

I think there needs to be some work on the clarity and wording. The phrase “despite overt differences in morphology” would be better expressed as “even when there are overt differences in morphology”. The original wording suggestions a contradiction, which is not necessarily the case, morphological comparisons can be done in morphologies with little disparity. The term “qualitative phenotypic differences” also feels inappropriate in this context; “phenotypic differences” is sufficient and avoids confusion with categorical traits.

The early paragraphs are somewhat disjointed. The movement from traditional measurements to modern geometric techniques is important but could be more tightly linked to the specific topic of volumetric morphometrics and registration-based pipelines, which is the paper’s actual focus. The discussion of the morphometric history presently includes an unnecessary detour to D’Arcy Thompson. While historically relevant, the connection to paper is less apparent. A more useful framing would be to (1) define linear morphometrics (i.e. callipers), (2) introduce geometric morphometrics, (3) mention algorithmic approaches such as LDDMM, ICP and polynomial-based descriptors (e.g. Euler characteristics), (4) state their downfalls, and then (5) link the need for volumetric and voxel-based approach of VBM and TBM. All these topics should be framed explicitly in relation to why a label-informed registration method is needed, thus aligning with the subsequent methodological developments in the paper.

The discussion around imaging modalities also is hard to follow. The current text refers to imaging advances without specifying modalities. Since the data involve micro-CT, you should introduce 3D imaging modalities (e.g. MRI, micro-CT) and connect these modalities to the challenge of generating accurate image registrations and then clarify how these fit into the ANTsX ecosystem.

The flow of the following paragraphs could also be improved. The discussion of VBM and TBM should include concrete examples with citations, ideally involving mouse development datasets (e.g. studies using 3D images for VBM comparisons) to demonstrate the current errors with intensity-only registration-based methods. The points made in paragraphs 4 and 5 contain related arguments and should be merged and reorganised to avoid redundancy. The discussion of limitations is informative but overly long. Condensing this section and restructuring it to lead directly into the introduction of the new anatomy-aware approach would greatly improve clarity.

Paragraphs 7-11 are also too long and interrupt the narrative. They contain useful information, but they need to be reduced and integrated more succinctly. Given that ANTsX is already well documented, the introduction should provide only the essential information and move quickly towards what the study novelly contributes.

The methods are generally clear and readable, but several key areas require clarification or expansion. The manuscript should explicitly describe how antsRegistration differs from labelImageRegistration. Is the difference solely the use of segmentation masks, or are there additional implementation differences? A high-level explanation of the label-informed cost function would also improve accessibility.

The authors use t-tests, but it is unclear whether normality was tested. Given that earlier results suggest poor performance of intensity-only registration, the underlying data distributions may deviate from normality. Normality tests should be reported, or a justification for parametric tests should be provided. Similar concerns apply to the Jacobian determinant data in the TBM analysis.

The TBM section needs more details. This includes (a) a formal definition of the Jacobian determinant, (b) an equation for its computation within this context and (c) a brief interpretation of what local expansion/ contraction signifies biologically. This information would make the section more accessible to readers not already familiar with TBM frameworks.

The PCA and inverse transforms section also requires some improvement. There needs to be a clearer explanation of what the inverse transformations represent, again preferably with an equation to back this up. There needs to be justification of why the range -500 to 500 was used in the scaling; did you test any alternatives?

In the first paragraph of the “Label-informed image registration improves Gli2-/- registration” in the Results, the text should include reference to Figure 3 to clarify what is being discussed.

Currently, the results include lots of qualitative comparisons of the data rather than quantitative comparisons. I feel these are needed to help substantiate lots of your claims. For example, to compare the improved correspondence using the label-based registration techniques, you could use the following techniques to quantify the overlaps: Dice similarity coefficient, Intersection over Union (IoU), Surface distance metrics and quantitative measures of scaling differences between registration modes. Without more quantitative backing, claims of improvement remain subjective and biased.

Figures 4-7 while conceptually useful, are difficult to interpret in their current form. These issues specifically are related to (a) insufficient clarity of heatmaps, (b) ambiguous alignment with corresponding input images and (c) limited explanation in captions. To improve these I would suggest: (1) placing the original labelled images in the first row, followed by the heatmaps, (2) use consistent colour palettes to label segments in these figures aligned with those in Figure 1 to further highlight which organs are being compared, which means adding segmentation labels to assist interpretation and (3) combining related figures of 5-7 to improve the interpretation. For Figure 4 you should explicitly state that the heatmaps compares the wildtype vs knockout differences. In this figure and following 5-7 I would change the plan; I would consider adding two columns showing the manual labels for the wildtype and knockout samples (with colour labels for organs) and then the two heatmap differences in the predictions done using the intensity-only and label-intensity approaches. There just requires increased guidance on what is being compared in Figure 4.

For the voxel number differences (i.e. 159,129 voxels) I would also assign percentages to these in terms of relation to the overall number of voxels.

Table 2 also conveys limited information as it currently only included manually generated data. To make table more useful could you: (1) include the intensity-only registration predicted volumes, (2) then indicate which correlations are statistically significant by highlighting them in bold and (3) providing more details on the differences in the predictions of the volumes between methods. I think this would help clarify the issue of volume predictions on the results. You could also consider doing a linear regression plot comparing the volumes generated across each of the methods and perform a Pearson or Spearman’s rank correlation comparison.

The comparison of the PCA space could also be substantially stronger with additional statistical analyses. You could for one fit a Gaussian mixture model (e.g. via mclust in R with n = 2) to evaluate how clustering would be assigned under a null and then compare this classification vs those obtained with the manually assigned values in the two different PCA spaces across the two methods. You should also evaluate the agreement between the intensity-only and label-guided methods numerically to show how much they differ. To do this you could use a combination of the Procrustean randomisation tests (PROTEST) to compare the matrix shapes, pairwise Euclidean distance comparisons between a selected point and the correlations between the PC scores. These would convert a subjective visual assessment into a robust statistical support.

Currently, the discussion has promising elements but lacks cohesion and sufficient referencing. There is need for additional references to substantiate claims. For instance, claims about the limitations about intensity-only registration and its issues with applying to developmental anatomy require references. There also needs to be references related to foundational VBM/TBM papers in its use. There should also be references about the ANTs/ANTsX methodology and any prior work of each of these techniques comparing knockout and wildtype developmental morphologies. This would help to support the issues with current methods.

Many points on the discussion rely on qualitative interpretation (e.g. “closer match”, “greater accuracy”). These statements must be substantiated quantitatively through the suggested metrics (Dice, IoU, PCA distances etc.).

The discussion around segmentation inputs is important, but the authors do not frame this within the context of their paper adequately. There are practical costs (i.e. time and accuracy) of using different methods to obtain segmentation labels, but their effects on the results of the TBM is not shown. It would be useful to provide a time comparison between the intensity and label-based registration techniques to see how much time manual labelling adds to the time. You could also test the feasibility of using semi-automated or automated segmentation methods of generating the label data required for the label-based method. A comparison of the manual vs automated label maps on the downstream results would be useful to backup the points made in this discussion section.

The structure of the discussion could also be improved. It would benefit from stronger linkage between the (a) methodological issue of previous methods, (b) the results of the study being used to demonstrate how they help overcome these issues and their limitations in addressing these issues, and (c) what are the broader implications of this? For instance, there needs to be greater discussion about the implications of being able to compare knockout and wildtypes through developmental stages. Additionally, the manuscript is missing a conclusion section. A short summary outlining the key findings and future directions would greatly improve readability and synthesis.

Figure 2 could be moved to the Supplementary material to make room for clearer comparative figures. Comparison of PC3/PC4 results could also be added to a supplemental.

The data is easily accessible via the provided GitHub link. Here the code for performing the analysis can also be found. I have not run the code due to the size of the outputs, but I am happy with the accessibility.

Overall, the study presents a promising and potentially valuable advance in registration-based morphometrics. The use of label-informed registration is conceptually strong and appears to offer meaningful improvements, particularly for anatomically challenging datasets such as malformed embryos. However, the manuscript requires substantial revisions to improve clarity, strengthen narrative flow, and most importantly provide quantitative evidence supporting claims of superiority over intensity-only registration. With additional analyses and improved cohesion, this paper has the potential to make a significant contribution to volumetric morphometric methodology.

Review can also be found in the .pdf file.

**********

what does this mean?). If published, this will include your full peer review and any attached files.

If you choose “no”, your identity will remain anonymous but your review may still be made public.

**Do you want your identity to be public for this peer review?**  For information about this choice, including consent withdrawal, please see our Privacy Policy

Reviewer #1: No

**********

[NOTE: If reviewer comments were submitted as an attachment file, they will be attached to this email and accessible via the submission site. Please log into your account, locate the manuscript record, and check for the action link "View Attachments". If this link does not appear, there are no attachment files.]

To ensure your figures meet our technical requirements, please review our figure guidelines: https://journals.plos.org/plosone/s/figures

You may also use PLOS’s free figure tool, NAAS, to help you prepare publication quality figures: https://journals.plos.org/plosone/s/figures#loc-tools-for-figure-preparation.

NAAS will assess whether your figures meet our technical requirements by comparing each figure against our figure specifications.

Attachment Submitted filename: Roston_et_al_PLOS_One_Review.pdf

# Author comment: Author response to Decision Letter 1

**DOI:** 10.1371/journal.pone.0357448.r003

2 Apr 2026

We thank the reviewer for their thoughtful comments and recommendations. We made substantial revisions to the manuscript to improve clarity, strengthen the narrative flow, and provide quantitative evidence supporting the claims. The revisions we made are detailed below.

Abstract

• We fixed typographic errors detailed by the reviewer

• The goal of the paper is to provide an example of applying a new registration method that has been added to the ANTs ecosystem and discuss its generic capabilities and applicability. Characterization of the Gli2 knockout phenotype was not the main goal of the manuscript, rather Gli2 knockout mice serve as an example of overt morphological differences that have impeded registration by other methods. The generalizations in the abstract referenced by the reviewer were intentional choices by the authors, aligned with the goal of the manuscript. To clarify our intended goal, wording was revised throughout the abstract and introduction.

o Reviewer’s comments: ““The abstract also feels a little over descriptive in terms of the general properties of ANTsC rather than specifically summarising the study’s findings. Sentences explaining how labels can be generated are too generic, the abstract should instead state which label were used in the present study and how they were obtained. Instead of just using the term knockout mice, I think the Gli2 gene should be explicitly mentioned in the Abstract rather than being first mentioned in the introduction. The term “downstream statistical analysis” is also too vague, the abstract should specify which analyses (e.g. volumetric comparisons, TBM, PCA). Overall, the abstract needs to be more focused on study-specific results and less on generic capabilities, so the readers can quickly understand what has been done and why it matters.“

Introduction

• We Incorporated wording suggested by the reviewer:

o “…even when there are overt differences in morphology”

o We changed “qualitative phenotypic differences” to “overt morphological differences” throughout the manuscript, and revised the manuscript for more consistent wording of “overt morphological differences”

Reviewer’s statement: “I think there needs to be some work on the clarity and wording. The phrase “despite overt differences in morphology” would be better expressed as “even when there are overt differences in morphology”. The original wording suggestions a contradiction, which is not necessarily the case, morphological comparisons can be done in morphologies with little disparity. The term “qualitative phenotypic differences” also feels inappropriate in this context; “phenotypic differences” is sufficient and avoids confusion with categorical traits.”

o Revised discussion of the evolution of morphometric techniques in accordance with reviewer suggestions:

Reviewer’s statements: “The early paragraphs are somewhat disjointed. The movement from traditional measurements to modern geometric techniques is important but could be more tightly linked to the specific topic of volumetric morphometrics and registration-based pipelines, which is the paper’s actual focus. The discussion of the morphometric history presently includes an unnecessary detour to D’Arcy Thompson. While historically relevant, the connection to paper is less apparent. A more useful framing would be to (1) define linear morphometrics (i.e. callipers), (2) introduce geometric morphometrics, (3) mention algorithmic approaches such as LDDMM, ICP and polynomial-based descriptors (e.g. Euler characteristics), (4) state their downfalls, and then (5) link the need for volumetric and voxel-based approach of VBM and TBM. All these topics should be framed explicitly in relation to why a label-informed registration method is needed, thus aligning with the subsequent methodological developments in the paper.”

• Reorganized the introduction of image registration and knockout mouse phenotyping, including adding sub-titles and streamlining the narrative:

o We added a new sub-title “Image Registration and revised and reorganized the text to:

Succinctly describe the “traditional” approach of intensity-only registration

Defined the concept of “anatomy-aware” approaches and briefly describe existing “anatomy-aware” methods

Explained how our anatomy-aware approach is different than existing methods and introduce it as new functionality in the ANTsX ecoystem

Reviewer’s statement: “The discussion around imaging modalities also is hard to follow. The current text refers to imaging advances without specifying modalities. Since the data involve micro-CT, you should introduce 3D imaging modalities (e.g. MRI, micro-CT) and connect these modalities to the challenge of generating accurate image registrations and then clarify how these fit into the ANTsX ecosystem.”

Reviewer’s statement: “The points made in paragraphs 4 and 5 contain related arguments and should be merged and reorganised to avoid redundancy. The discussion of limitations is informative but overly long. Condensing this section and restructuring it to lead directly into the introduction of the new anatomy-aware approach would greatly improve clarity. Paragraphs 7-11 are also too long and interrupt the narrative. They contain useful information, but they need to be reduced and integrated more succinctly. Given that ANTsX is already well documented, the introduction should provide only the essential information and move quickly towards what the study novelly contributes.”

o We added text to better explain the role of image registration in mouse phenotyping and our goal of using mouse phenotyping as an example for the use of label-informed image registration. We added text to explain the need for high-throughput, automated phenotyping pipelines for reverse genetic screens, the key role for image registration in proposed analytical pipelines, the use of TBM in these pipelines, and the limitations of “traditional” intensity-only registration for this application. TBM is that it is a common technique described in detail elsewhere and was used in this paper because it is commonly used in the phenotype analysis pipelines for knockout mice on which we based this manuscript.

Reviewer comment: “The flow of the following paragraphs could also be improved. The discussion of VBM and TBM should include concrete examples with citations, ideally involving mouse development datasets (e.g. studies using 3D images for VBM comparisons) to demonstrate the current errors with intensity-only registration-based methods.”

Methods

• We added a new section explaining the labelImageRegistration method in greater detail

o Reviewer comment: “The methods are generally clear and readable, but several key areas require clarification or expansion. The manuscript should explicitly describe how antsRegistration differs from labelImageRegistration. Is the difference solely the use of segmentation masks, or are there additional implementation differences? A high-level explanation of the label-informed cost function would also improve accessibility.”

• We changed the statistical test for organ volume data to non-parametric Mann-Whitney U tests instead of t-tests. To better visualize the distributions of organ volume measurements, we also added a box and whiskers plot of organ volumes. We added clarification in the text that our main goal for including volumetric measurements is as a ground-truth to which to compare TBM results. For the TBM analysis, we utilized a voxel-wise linear regression under the framework of the General Linear Model (GLM), which is the standard, well-established approach for voxel- and tensor-based morphometry (Ashburner & Friston, 2000). While the GLM does carry an assumption of normally distributed errors, prior methodological work has demonstrated that voxel-wise group comparisons in balanced or nearly balanced designs are highly robust to violations of normality (Salmond et al., 2002). Therefore, we have retained the GLM/linear regression approach for the TBM analysis. However, we realized that we inadvertently referred to this as a 't-test' in the Figure 4 caption and the Results text, which caused understandable confusion. We have corrected these typos throughout the manuscript to accurately reflect the use of linear regression.

o Reviewer comment: “The authors use t-tests, but it is unclear whether normality was tested. Given that earlier results suggest poor performance of intensity-only registration, the underlying data distributions may deviate from normality. Normality tests should be reported, or a justification for parametric tests should be provided. Similar concerns apply to the Jacobian determinant data in the TBM analysis.”

• We added text to better explain the Jacobian determinant in this context, the steps of TBM, and the biological interpretation of TBM results

o Reviewer comment: “The TBM section needs more details. This includes (a) a formal definition of the Jacobian determinant, (b) an equation for its computation within this context and (c) a brief interpretation of what local expansion/ contraction signifies biologically. This information would make the section more accessible to readers not already familiar with TBM frameworks.”

• We added text to better explain the application and interpretation of PCA in this context, the decision to use inverse transforms in this analysis, and the choice of the scaling range -500 to 500. This scaling does not alter the direction of the eigenvectors. It simply increases or decreases the magnitude of the transformations to make the morphological changes along the PC axes visually discernible without introducing unrealistic distortions or clipping.

• We added a section on Dice calculations

• We revised section on visualizations for clarity

Results

• Text was changed throughout the Results section to clarify the finding that that morphometrics resulting from intensity-only registration did not reflect real, observable morphological differences and that only label-informed registration was able to align knockout images with the template such that the morphometrics reflect observable morphological differences:

o This finding was clarified by the incorporation of Dice scores in Figure 2, text regarding overlaid images, and the text explaining Figures 4, 5-8

o We thank the reviewer for suggesting methods to more deeply interrogate the PCA spaces. While statistical methods such as Gaussian mixture models and PROTEST are excellent approaches for comparing morphospaces in typical evolutionary or developmental studies, we have opted not to perform these statistical comparisons here because doing so would misrepresent the nature of our data. The fundamental basis of the PCA in this context is the output of the deformable registration. As demonstrated by multiple lines of evidence, including the Dice statistics and visual overlays, the intensity-only registration fundamentally failed to accurately align the knockout subjects. Consequently, the PCA space derived from the intensity-only registration does not represent a valid biological morphospace; rather, it represents registration error. Applying robust statistical comparisons between the two PCA spaces would falsely imply that both registration methods produced valid morphometric results that can be meaningfully benchmarked against one another, which our data clearly show is not the case.

Reviewer comment: “The comparison of the PCA space could also be substantially stronger with additional statistical analyses. You could for one fit a Gaussian mixture model (e.g. via mclust in R with n = 2) to evaluate how clustering would be assigned under a null and then compare this classification vs those obtained with the manually assigned values in the two different PCA spaces across the two methods. You should also evaluate the agreement between the intensity-only and label-guided methods numerically to show how much they differ. To do this you could use a combination of the Procrustean randomisation tests (PROTEST) to compare the matrix shapes, pairwise Euclidean distance comparisons between a selected point and the correlations between the PC scores. These would convert a subjective visual assessment into a robust statistical support.”

• Overall structure of the Results section and sub-titles were revised for clarity and revised figure references

o Reviewer’s comment: “In the first paragraph of the “Label-informed image registration improves Gli2-/- registration” in the Results, the text should include reference to Figure 3 to clarify what is being discussed.”

• We added a section on Dice coefficients (a new plot and text) as a quantitative measure of registration accuracy

o Reviewer’s comment: “Currently, the results include lots of qualitative comparisons of the data rather than quantitative comparisons. I feel these are needed to help substantiate lots of your claims. For example, to compare the improved correspondence using the label-based registration techniques, you could use the following techniques to quantify the overlaps: Dice similarity coefficient, Intersection over Union (IoU), Surface distance metrics and quantitative measures of scaling differences between registration modes. Without more quantitative backing, claims of improvement remain subjective and biased.”

• Table 2 was removed and a new figure was added showing box and whisker plots for organ volumes to allow better visualization of the mean and variance of organ volumes. Text was added and revised to clarify that these measurements were included as a ground-truth for the TBM results. Because the Dice statistic for intensity-only registration was so low, we do not think it is useful to show predicted volumes (which would be inaccurate). Rather, we added text to clarify that the organ volumes calculated from manual segmentations provide a ground-truth to which to compare the TBM results (i.e., the TBM results are consistent with measured organ volume differences)

o Reviewer comments: “Table 2 also conveys limited information as it currently only included manually generated data. To make table more useful could you: (1) include the intensity-only registration predicted volumes, (2) then indicate which correlations are statistically significant by highlighting them in bold and (3) providing more details on the differences in the predictions of the volumes between methods. I think this would help clarify the issue of volume predictions on the results. You could also consider doing a linear regression plot comparing the volumes generated across each of the methods and perform a Pearson or Spearman’s rank correlation comparison.”

• Figures 4-7 (now Figures 4, 6-8) were revised based on the reviewer’s recommendations. Figure 4 now includes an additional column showing an example knockout subject and Figures 6-8 (formerly 5-7) were revised so that example wildtype and knockout subjects are in the top row. Organ labels matching Figure 1 were added to Figures 6-8 for additional clarity.

o Reviewer’s comment: “Figures 4-7 while conceptually useful, are difficult to interpret in their current form. These issues specifically are related to (a) insufficient clarity of heatmaps, (b) ambiguous alignment with corresponding input images and (c) limited explanation in captions. To improve these I would suggest: (1) placing the original labelled images in the first row, followed by the heatmaps, (2) use consistent colour palettes to label segments in these figures aligned with those in Figure 1 to further highlight which organs are being compared, which means adding segmentation labels to assist interpretation and (3) combining related figures of 5-7 to improve the interpretation. For Figure 4 you should explicitly state that the heatmaps compares the wildtype vs knockout differences. In this figure and following 5-7 I would change the plan; I would consider adding two columns showing the manual labels for the wildtype and knockout samples (with colour labels for organs) and then the two heatmap differences in the predictions done using the intensity-only and label-intensity approaches. There just requires increased guidance on what is being compared in Figure 4.”

• Percentages were added to the voxel number differences for TBM

o Reviewer comment: “For the vo

Attachment Submitted filename: LabelIntensityRegistrationPaper_v2_response.docx

# Peer review: Decision Letter 1

**DOI:** 10.1371/journal.pone.0357448.r004

**Authors:** Alpen Ortug

15 Jun 2026

PLOS One

Dear Dr. Maga,

Thank you for submitting your manuscript to PLOS ONE. After careful consideration, we feel that it has merit but does not fully meet PLOS ONE’s publication criteria as it currently stands. Therefore, we invite you to submit a revised version of the manuscript that addresses the points raised during the review process.

Thank you for submitting your manuscript to PLOS ONE. We have now received the comments from the second reviewer.

Please see below for the comments from both reviewers. While Reviewer 1's concerns have largely been addressed, Reviewer 2 has raised several substantive issues that require further revision.

After careful consideration, we believe that the manuscript has merit and remains of interest to the journal. However, in light of the additional concerns raised by Reviewer 2, the manuscript does not yet fully meet PLOS ONE's publication criteria. Therefore, we invite you to undertake a further major revision and submit a revised version of the manuscript that addresses the comments from both reviewers, together with a detailed point-by-point response.

Please submit your revised manuscript by Jul 30 2026 11:59PM. If you will need more time than this to complete your revisions, please reply to this message or contact the journal office at plosone@plos.org. When you're ready to submit your revision, log on to https://www.editorialmanager.com/pone/ and select the 'Submissions Needing Revision' folder to locate your manuscript file.

- A letter that responds to each point raised by the academic editor and reviewer(s). You should upload this letter as a separate file labeled 'Response to Reviewers'.
- A marked-up copy of your manuscript that highlights changes made to the original version. You should upload this as a separate file labeled 'Revised Manuscript with Track Changes'.
- An unmarked version of your revised paper without tracked changes. You should upload this as a separate file labeled 'Manuscript'.

If you would like to make changes to your financial disclosure, please include your updated statement in your cover letter. Guidelines for resubmitting your figure files are available below the reviewer comments at the end of this letter.

If applicable, we recommend that you deposit your laboratory protocols in protocols.io to enhance the reproducibility of your results. Protocols.io assigns your protocol its own identifier (DOI) so that it can be cited independently in the future. For instructions see: https://journals.plos.org/plosone/s/submission-guidelines#loc-laboratory-protocols. Additionally, PLOS ONE offers an option for publishing peer-reviewed Lab Protocol articles, which describe protocols hosted on protocols.io. Read more information on sharing protocols at https://plos.org/protocols?utm_medium=editorial-email&utm_source=authorletters&utm_campaign=protocols.

As the corresponding author, your ORCID iD is verified in the submission system and will appear in the published article. PLOS supports the use of ORCID, and we encourage all coauthors to register for an ORCID iD and use it as well. Please encourage your coauthors to verify their ORCID iD within the submission system before final acceptance, as unverified ORCID iDs will not appear in the published article. *Only*  the individual author can complete the verification step; PLOS staff *cannot*  verify ORCID iDs on behalf of authors.

We look forward to receiving your revised manuscript.

Kind regards,

Alpen Ortug, PhD

Academic Editor

PLOS One

Journal Requirements:

If the reviewer comments include a recommendation to cite specific previously published works, please review and evaluate these publications to determine whether they are relevant and should be cited. There is no requirement to cite these works unless the editor has indicated otherwise.

[Note: HTML markup is below. Please do not edit.]

Reviewers' comments:

Reviewer's Responses to Questions

**Comments to the Author**

Reviewer #1: All comments have been addressed

Reviewer #2: (No Response)

**********

2. Is the manuscript technically sound, and do the data support the conclusions?

Reviewer #1: Yes

Reviewer #2: Yes

**********

3. Has the statistical analysis been performed appropriately and rigorously?

Reviewer #1: Yes

Reviewer #2: Yes

**********

4. Have the authors made all data underlying the findings in their manuscript fully available?

The PLOS Data policy

Reviewer #1: Yes

Reviewer #2: Yes

**********

5. Is the manuscript presented in an intelligible fashion and written in standard English?

Reviewer #1: Yes

Reviewer #2: Yes

**********

Reviewer #1: The authors have satisfactory addressed the concerns raised in the initial review or have provided adequate justification where changes were not implemented. Prior to publication, however, several minor issues should be addressed to further improve the manuscript:

1. There is an extraneous space at the beginning of the third section of the Image Registration section of the Introduction.

2. In the first sentence of the fourth paragraph of the Automated Phenotyping of Genetic Knockouts, the word “have” in “we have selected a genetic” is not needed and can be removed.

3. The final paragraph of the Introduction reads more like a conclusion. Given the substantial length of the introduction, its inclusion may not be necessary. If retained, it should be substantially reduced. Alternatively, a better option may be to revise this and incorporate it into a dedicated concluding section, which is currently lacking. This will help to provide a more holistic view of the overall study.

4. The results presented in Table 2 could be integrated into Figure 5. Specifically, the W and p-values could be included within each organ-specific panel, thereby eliminating the need for Table 2. Additionally, the Figure 5 caption is currently too brief and should be expanded. At the bare minimum this should be to define the abbreviation of “NS” and to provide a clearer summary of the results shown.

5. In Figure 1, please verify that all organ labels are clearly visible. For example, labels “6” and “7” are not readily apparent, and the label “1” on the coronal slice of the second panel is difficult to discern.

6. Figure 2 is informative but could be improved. If the boxplot format is retained, consider adding a line indicating the mean value across all organs within each panel. However, a more effective alternative may be to present combined regression plots comparing values obtained from the two registration methods (i.e. Intensity-only vs Label-Informed) for both WT and KO datasets. Here the confidence intervals and labels for each individual organ can still be added into the plot.

7. In Figure 3, the yellow color label appears somewhat orange; a more clearly distinguishable color may help improve clarity. Additionally, ensure that the text labels match the corresponding colors used in the figure. To improve readability, consider adding headings above each of the four panel groups to indicate the associated transformations (Combined forward transforms vs linear transforms only).

8. Figures 6-8 may be excessive for the main text and could be moved to the supplementary material. Many of the patterns they illustrate cane be observed in Figure 4 upon closer inspection.

Reviewer #2: Authors developed a registration method by incorporating parcellated label maps into the cost function. Registration is mostly performed using image intensities. In this application, authors proposed to use expert guided segmentations of organs such as brain, spinal cord, liver, and heart from Micro-CT scans of wild type and Gli2 knockout embryos. Authors showed that their proposed approach (combination of label maps and image intensities), compared to intensity based registration, substantially improved registration by showing dice overlap statistics and qualitative examples. In addition, authors showed that accurate derivation of warps to template image provides better quantification of morphological differences between wild type and Gli2 knockout embryos. Provided heat maps derived from voxel based morphometry and plots of principal component analysis are used to demonstrate qualitative and quantitative differences between wild type and Gli2 knockout embryos. Authors generated a template image by averaging 35 wildtype micro-CT scans from 8 inbred and outbred knockout mouse lines. Over 250 embryos collected and scanned for an ongoing reverse genetic screen. Gli2 was selected for this particular study. I have listed my suggestions below:

-Paper is well organized but needs some modifications and clarifications.

-Some references cited several times throughout the manuscript, such as 9, and 32. Some of these repeated citations are unnecessary, I suggest these should be reviewed for necessity. In addition, I see references to books or workshop proceedings, if there are specific parts in this book or proceedings that would be more relevant for the reader to check in case of a need.

“phenotype discovery described by Wong et al. (2014), Dickinson et al. (2016), and

Horner et al. (2021) (32,35,51).” Here, Author/Year and Number references are not in order.

I think authors should emphasize that their contribution is to develop this labelImageRegistration function as an additional functionality in Ants package. While reading the manuscript I thought this is already available in the package and someone else developed these functions, I also needed to check the website to notice that this is not coming with default installation.

In the methods section, the E_total equation is given, but w_i is not described in the same sentence, it is not clear how these weights (w_i and w_intensity) are calculated or initialized.

In the Embryo Collection and Micro-CT section: It is specified that these subjects were a subset of over 250 embryos, that would be good if authors can explain why we don’t read a certain number here. How many of these embryos were used in the experiments? This is not clear.

In the Registration section: “Subject images were also rigidly aligned with the template prior to this study to support easy and rapid visualization of subject anatomy in standard anatomical planes.” I think this is also a required step prior to nonlinear warp estimation, that would also be mentioned as a support, this is a suggestion.

I think there are a lot of subsections in Methods, such as the Organ Volume Analysis section. This section is not clear and I don’t understand why Mann-Whitney U tests mentioned here, what it compares should be mentioned clearly.

Results section: “Missing and misshapen vertebrae have been previously described in Gli2-/- mouse embryos (56,64) but the effects of these defects on overall geometry of the vertebral column and morphometrics of the embryo have not been previously studied.” I think this sentence shouldn’t appear in the results, and sentences exist in the introduction, if 56 and 64 are not cited in the introduction that would be good to move this into the introduction section.

In section “Dice Overlap between manual and registration-based labels”: I think authors may give how the overall mean dice statistics calculated, when I look at Figure 2 and whisker box plots of intensity only registration of knockout subjects I would expect to have a mean value smaller than 0.81, but I’m not sure about this suggestion. Please consider this as a minor note to authors to double check.

In section “Overlay of knockouts transformed into template space over template”: I think an additional comment needed for Figure 3C, where I see yellow region above region iv not warped correctly, is that an insignificant region for the investigation?

----I see the figure legends do not match what is being described in this section: in the last paragraph here Figure 3E,F references for knockout subjects, but in the figure F is for wild type; Figure 3G,H referenced for deformable registration, but they are for linear registration. Figure 3E,G referenced for label-intensity but in fact it is not the case I see in the figures.

In the section “Label-Informed TBM yields greater statistical power”: First paragraph is a repetition, I think that is mentioned in the methods section. I suggest removal of this part.

----“TBM using label-informed image registration identified 50% more significantly different Jacobian values than TBM using intensity-only registration” Is 50% an approximate value? If the exact value is available that would be mentioned here.

Table 2: Caption is not clear, W is not described. Please highlight/mark the p-values which are annotated with single or multiple asterisks in Figure 5. The legends of asterisks are also missing in Figure 5.

In section “Label-informed TBM reflects overt gross morphological differences”:

----“this displacement is also associated with a diaphragmatic hernia (Figure 6).” I don’t see any caption information regarding the hernia in Figure 6, that would be great if the hernia is marked in Figure 6.

----“which is readily observable morphology in the knockout embryos.” I think this sentence needs a citation.

----“A similar phenomenon is also observed in the spinal cord.” I think this sentence is not clear, so it would be good to refer to the similar observations again.

Figure 8C,D: please mention the description of the background image.

In section “Label-informed PCA captures genotype-associated phenotypes”:

----First sentence: we don’t need to define PCA again, already mentioned in the methods section.

----“Even though there are obvious and highly penetrant phenotypic differences in knockout and wildtype subjects, such as severe scoliosis (see Figure 1, Suppl. Figure 1),” Why this sentence is not referring to Figure 7, which is about scoliosis.

----“these include: differences in the subjects’ spinal curvature (i.e., scoliosis), an increase in heart ventricle volume, differences in head shape and orientation, and a reduction in the space between the bladder and rectum (associated with a vesicorectal fistula).” I think this sentence should be part of the discussion, it is not good to read this in the results section.

Figure 9: Caption has a reference to Fig 1, this is not good, caption should only describe what is given in the figure.

Reference 22, check if this is properly cited in the manuscript, I couldn’t locate, but maybe I just missed.

**********

what does this mean?). If published, this will include your full peer review and any attached files.

If you choose “no”, your identity will remain anonymous but your review may still be made public.

**Do you want your identity to be public for this peer review?**  For information about this choice, including consent withdrawal, please see our Privacy Policy

Reviewer #1: No

Reviewer #2: No

**********

[NOTE: If reviewer comments were submitted as an attachment file, they will be attached to this email and accessible via the submission site. Please log into your account, locate the manuscript record, and check for the action link "View Attachments". If this link does not appear, there are no attachment files.]

To ensure your figures meet our technical requirements, please review our figure guidelines: https://journals.plos.org/plosone/s/figures

You may also use PLOS’s free figure tool, NAAS, to help you prepare publication quality figures: https://journals.plos.org/plosone/s/figures#loc-tools-for-figure-preparation.

NAAS will assess whether your figures meet our technical requirements by comparing each figure against our figure specifications.

# Author comment: Author response to Decision Letter 2

**DOI:** 10.1371/journal.pone.0357448.r005

27 Jul 2026

We thank the reviewers for their thoughtful comments and suggestions.

Reviewer #1: The authors have satisfactory addressed the concerns raised in the initial review or have provided adequate justification where changes were not implemented. Prior to publication, however, several minor issues should be addressed to further improve the manuscript:

1. There is an extraneous space at the beginning of the third section of the Image Registration section of the Introduction.

We fixed a typo of a double space at the beginning of a sentence, but did not notice any other extraneous space.

2. In the first sentence of the fourth paragraph of the Automated Phenotyping of Genetic Knockouts, the word “have” in “we have selected a genetic” is not needed and can be removed.

Removed the word “have”

3. The final paragraph of the Introduction reads more like a conclusion. Given the substantial length of the introduction, its inclusion may not be necessary. If retained, it should be substantially reduced. Alternatively, a better option may be to revise this and incorporate it into a dedicated concluding section, which is currently lacking. This will help to provide a more holistic view of the overall study.

We thank the reviewer for this comment. For clarity, the authors prefer to provide a preview of the results and conclusions at the end of the introduction. Doing so helps orient the reader as they read the Methods, Results, Discussion. Many readers read scientific papers out of the order in which they were written, so we believe providing guide posts (even if slightly repetitive) in different sections helps with clarity.

4. The results presented in Table 2 could be integrated into Figure 5. Specifically, the W and p-values could be included within each organ-specific panel, thereby eliminating the need for Table 2. Additionally, the Figure 5 caption is currently too brief and should be expanded. At the bare minimum this should be to define the abbreviation of “NS” and to provide a clearer summary of the results shown.

The information from Table 2 was added to Figure 5 and Table 2 was removed.

5. In Figure 1, please verify that all organ labels are clearly visible. For example, labels “6” and “7” are not readily apparent, and the label “1” on the coronal slice of the second panel is difficult to discern.

Edited Figure 1 to add labels 7 and 8 and moved label 1 to the left of the embryo

6. Figure 2 is informative but could be improved. If the boxplot format is retained, consider adding a line indicating the mean value across all organs within each panel. However, a more effective alternative may be to present combined regression plots comparing values obtained from the two registration methods (i.e. Intensity-only vs Label-Informed) for both WT and KO datasets. Here the confidence intervals and labels for each individual organ can still be added into the plot.

A dotted line indicating the mean Dice statistic was added to each panel.

7. In Figure 3, the yellow color label appears somewhat orange; a more clearly distinguishable color may help improve clarity. Additionally, ensure that the text labels match the corresponding colors used in the figure. To improve readability, consider adding headings above each of the four panel groups to indicate the associated transformations (Combined forward transforms vs linear transforms only).

Added labels for the associated transformations. Lightened the yellow text to increase contrast against the black background. The figures are true yellow and cyan, but appear as different shades in part due to reduced opacity against the black background.

8. Figures 6-8 may be excessive for the main text and could be moved to the supplementary material. Many of the patterns they illustrate cane be observed in Figure 4 upon closer inspection.

The TBM results are the same in Figure 4 and in Figures 6-8, but Figures 6-8 are meant to highlight how the TBM results correspond with actual morphological differences. The purpose of these figures is to make the main text more accessible to readers who may not be as familiar with all of the anatomical terms we use by providing a visual representation and to allow readers with more background in anatomy to independently assess the anatomy shown in the images rather than relying solely on our descriptions. We therefore think these figures are integral to the manuscript and prefer not to move them to the supplementary material.

Reviewer #2: Authors developed a registration method by incorporating parcellated label maps into the cost function. Registration is mostly performed using image intensities. In this application, authors proposed to use expert guided segmentations of organs such as brain, spinal cord, liver, and heart from Micro-CT scans of wild type and Gli2 knockout embryos. Authors showed that their proposed approach (combination of label maps and image intensities), compared to intensity based registration, substantially improved registration by showing dice overlap statistics and qualitative examples. In addition, authors showed that accurate derivation of warps to template image provides better quantification of morphological differences between wild type and Gli2 knockout embryos. Provided heat maps derived from voxel based morphometry and plots of principal component analysis are used to demonstrate qualitative and quantitative differences between wild type and Gli2 knockout embryos. Authors generated a template image by averaging 35 wildtype micro-CT scans from 8 inbred and outbred knockout mouse lines. Over 250 embryos collected and scanned for an ongoing reverse genetic screen. Gli2 was selected for this particular study. I have listed my suggestions below:

-Paper is well organized but needs some modifications and clarifications.

-Some references cited several times throughout the manuscript, such as 9, and 32. Some of these repeated citations are unnecessary, I suggest these should be reviewed for necessity. In addition, I see references to books or workshop proceedings, if there are specific parts in this book or proceedings that would be more relevant for the reader to check in case of a need.

“phenotype discovery described by Wong et al. (2014), Dickinson et al. (2016), and

Horner et al. (2021) (32,35,51).” Here, Author/Year and Number references are not in order.

This is because of the citation management software. We will ensure that the orders match in the publication.

I think authors should emphasize that their contribution is to develop this labelImageRegistration function as an additional functionality in Ants package. While reading the manuscript I thought this is already available in the package and someone else developed these functions, I also needed to check the website to notice that this is not coming with default installation.

The second author of this manuscript, Nick Tustison, is one of the original developers of ANTs. The additional functionality of label-image registration was developed by Dr. Tustinson and has already been integrated into ANTsR and ANTsPy (e.g., https://github.com/ANTsX/ANTsPy/pull/743). This manuscript is the first presentation and demonstration of the method, its utility, and its application to a biological problem in the literature. Label-image registration functionality is automatically included with more recent versions of ANTsR and ANTsPy. To clarify these points, we revised the text at the beginning of the Methods section to emphasize that this is new functionality. There was already sentence in the intro that states that, but we now restate it in the Methods for clarity.

In the methods section, the E_total equation is given, but w_i is not described in the same sentence, it is not clear how these weights (w_i and w_intensity) are calculated or initialized.

We added text to clarify this

In the Embryo Collection and Micro-CT section: It is specified that these subjects were a subset of over 250 embryos, that would be good if authors can explain why we don’t read a certain number here. How many of these embryos were used in the experiments? This is not clear.

“Over 250 embryos” was used because collection and scanning was ongoing, so the precise number was changing. We have updated the number to reflect the current total number of scans.

In the Registration section: “Subject images were also rigidly aligned with the template prior to this study to support easy and rapid visualization of subject anatomy in standard anatomical planes.” I think this is also a required step prior to nonlinear warp estimation, that would also be mentioned as a support, this is a suggestion.

Yes, rigid alignment is one of the components of the linear registration we use in this manuscript. The code we provide in the Github repository assumes that subjects are already in an approximately standardized orientation. If someone were to try to adapt our code to another dataset, they may find it doesn’t work well if their subjects initially have very different orientations. So, we included this sentence to clarify that there was a standardization step that was done previously and not included in this study or in the code provided in the Github repository. (The prior rigid registration is obvious if one opens the scan images provided in the repository, but we thought it was useful to state explicitly that it was a prior step because people may not visualize the images when accessing our code.)

I think there are a lot of subsections in Methods, such as the Organ Volume Analysis section. This section is not clear and I don’t understand why Mann-Whitney U tests mentioned here, what it compares should be mentioned clearly.

We revised that section as suggested.

Results section: “Missing and misshapen vertebrae have been previously described in Gli2-/- mouse embryos (56,64) but the effects of these defects on overall geometry of the vertebral column and morphometrics of the embryo have not been previously studied.” I think this sentence shouldn’t appear in the results, and sentences exist in the introduction, if 56 and 64 are not cited in the introduction that would be good to move this into the introduction section.

This sentence was moved to the Introduction and removed from the results.

In section “Dice Overlap between manual and registration-based labels”: I think authors may give how the overall mean dice statistics calculated, when I look at Figure 2 and whisker box plots of intensity only registration of knockout subjects I would expect to have a mean value smaller than 0.81, but I’m not sure about this suggestion. Please consider this as a minor note to authors to double check.

The means we originally presented were calculated from the overall means of the registrations (i.e., calculated from the Dice statistic for all of the labels from a single individual subject, the “All” label in the plot in Figure 5), which we referred to as the “overall Dice statistic.” However, this is inconsistent with how we chose to present the data in Figure 5. So, for clarity and consistency, we now present the mean of the Dice statistics for all of the organ labels, not separated by individual registration.

In section “Overlay of knockouts transformed into template space over template”: I think an additional comment needed for Figure 3C, where I see yellow region above region iv not warped correctly, is that an insignificant region for the investigation?

The yellow region above iv is the tail. It was not of interest in this study because the position varies considerably and the variation in positioning is random and not a “structural” difference. We did not have a label in that region, but the alignment of the tail could be improved by including a label there. We have added text to the figure legend to explain this result.

----I see the figure legends do not match what is being described in this section: in the last paragraph here Figure 3E,F references for knockout subjects, but in the figure F is for wild type; Figure 3G,H referenced for deformable registration, but they are for linear registration. Figure 3E,G referenced for label-intensity but in fact it is not the case I see in the figures.

Revised the figure references

In the section “Label-Informed TBM yields greater statistical power”: First paragraph is a repetition, I think that is mentioned in the methods section. I suggest removal of this part.

----“TBM using label-informed image registration identified 50% more significantly different Jacobian values than TBM using intensity-only registration” Is 50% an approximate value? If the exact value is available that would be mentioned here.

Table 2: Caption is not clear, W is not described. Please highlight/mark the p-values which are annotated with single or multiple asterisks in Figure 5. The legends of asterisks are also missing in Figure 5.

W-statistic

In section “Label-informed TBM reflects overt gross morphological differences”:

----“this displacement is also associated with a diaphragmatic hernia (Figure 6).” I don’t see any caption information regarding the hernia in Figure 6, that would be great if the hernia is marked in Figure 6.

Figure 6 does not show one of the embryos with a hernia. The in-text figure references were revised to clarify this.

----“which is readily observable morphology in the knockout embryos.” I think this sentence needs a citation.

Added Figure reference

----“A similar phenomenon is also observed in the spinal cord.” I think this sentence is not clear, so it would be good to refer to the similar observations again.

Figure 8C,D: please mention the description of the background image.

Slice planes and “overlaid onto the template image” were added to the legends of Figure 6-8 for clarity.

In section “Label-informed PCA captures genotype-associated phenotypes”:

----First sentence: we don’t need to define PCA again, already mentioned in the methods section.

----“Even though there are obvious and highly penetrant phenotypic differences in knockout and wildtype subjects, such as severe scoliosis (see Figure 1, Suppl. Figure 1),” Why this sentence is not referring to Figure 7, which is about scoliosis.

Added Figure 7. In an older version of the manuscript the PCA results were presented prior to what are now Figures 6-8 and we overlooked this reference in the revision.

----“these include: differences in the subjects’ spinal curvature (i.e., scoliosis), an increase in heart ventricle volume, differences in head shape and orientation, and a reduction in the space between the bladder and rectum (associated with a vesicorectal fistula).” I think this sentence should be part of the discussion, it is not good to read this in the results section.

This sentence is included in the Results section because it specifically describes the morphological differences that result from transforming the template along the PC2 axis: “Transformation of the template image along PC2 shows that morphological differences along PC2…” Following this sentence, we explain the morphological differences observed along the PC1 axis.

Figure 9: Caption has a reference to Fig 1, this is not good, caption should only describe what is given in the figure.

Removed the reference.

Reference 22, check if this is properly cited in the manuscript, I couldn’t locate, but maybe I just missed.

Reference 22 is listed in the introduction in the range of 20-23:

“These methods have been applied to wide variety of study subjects, from plant leaves to vertebrate skulls, and have been applied to a wide variety of biological questions from embryonic development to biological variation to evolution (8,20–23)”

Attachment Submitted filename: LabelIntensityRegistrationPaper_v3_rebuttal.docx

# Peer review: Decision Letter 2

**DOI:** 10.1371/journal.pone.0357448.r006

**Authors:** Alpen Ortug

19 Aug 2026

Anatomy-aware, label-informed approach improves image registration for challenging datasets

PONE-D-25-51789R2

Dear Dr. Maga,

We’re pleased to inform you that your manuscript has been judged scientifically suitable for publication and will be formally accepted for publication once it meets all outstanding technical requirements.

Within one week, you’ll receive an e-mail detailing the required amendments. When these have been addressed, you’ll receive a formal acceptance letter and your manuscript will be scheduled for publication.

An invoice will be generated when your article is formally accepted. Please note, if your institution has a publishing partnership with PLOS and your article meets the relevant criteria, all or part of your publication costs will be covered. Please make sure your user information is up-to-date by logging into Editorial Manager at Editorial Manager® and clicking the ‘Update My Information' link at the top of the page. For questions related to billing, please contact billing support.

If your institution or institutions have a press office, please notify them about your upcoming paper to help maximize its impact. If they’ll be preparing press materials, please inform our press team as soon as possible -- no later than 48 hours after receiving the formal acceptance. Your manuscript will remain under strict press embargo until 2 pm Eastern Time on the date of publication. For more information, please contact onepress@plos.org.

Kind regards,

Alpen Ortug, PhD

Academic Editor

PLOS One

Additional Editor Comments (optional):

Thank you for carefully addressing the reviewers’ comments and revising the manuscript accordingly.

Please also see Reviewer 2’s final minor suggestion regarding Figure 5 (p-values greater than 0.05 should not be displayed in the figure; instead, only asterisks should be used to indicate statistically significant results, as described in the figure caption).

Reviewers' comments:

Reviewer's Responses to Questions

**Comments to the Author**

Reviewer #2: (No Response)

**********

2. Is the manuscript technically sound, and do the data support the conclusions?

Reviewer #2: Yes

**********

3. Has the statistical analysis been performed appropriately and rigorously?

Reviewer #2: Yes

**********

4. Have the authors made all data underlying the findings in their manuscript fully available?

The PLOS Data policy

Reviewer #2: Yes

**********

5. Is the manuscript presented in an intelligible fashion and written in standard English?

Reviewer #2: Yes

**********

Reviewer #2: All of my initial concerns addressed by the authors. I have just a minor suggestion about Figure 5: I think p-values which are greater than 0.05 should not be printed on the image as a legend, I suggest using only asterisks as legends in order to mark the significance as given in the caption, if there is no star it means p>0.05.

**********

what does this mean?). If published, this will include your full peer review and any attached files.

If you choose “no”, your identity will remain anonymous but your review may still be made public.

**Do you want your identity to be public for this peer review?**  For information about this choice, including consent withdrawal, please see our Privacy Policy

Reviewer #2: No

**********

# Editor report: Acceptance letter

**DOI:** 10.1371/journal.pone.0357448.r007

**Authors:** Alpen Ortug

PONE-D-25-51789R2

PLOS One

Dear Dr. Maga,

I'm pleased to inform you that your manuscript has been deemed suitable for publication in PLOS One. Congratulations! Your manuscript is now being handed over to our production team.

At this stage, our production department will prepare your paper for publication. This includes ensuring the following:

* All references, tables, and figures are properly cited

* All relevant supporting information is included in the manuscript submission,

* There are no issues that prevent the paper from being properly typeset

You will receive further instructions from the production team, including instructions on how to review your proof when it is ready. Please keep in mind that we are working through a large volume of accepted articles, so please give us a few days to review your paper and let you know the next and final steps.

Lastly, if your institution or institutions have a press office, please let them know about your upcoming paper now to help maximize its impact. If they'll be preparing press materials, please inform our press team within the next 48 hours. Your manuscript will remain under strict press embargo until 2 pm Eastern Time on the date of publication. For more information, please contact onepress@plos.org.

You will receive an invoice from PLOS for your publication fee after your manuscript has reached the completed accept phase. If you receive an email requesting payment before acceptance or for any other service, this may be a phishing scheme. Learn how to identify phishing emails and protect your accounts at https://explore.plos.org/phishing.

If we can help with anything else, please email us at customercare@plos.org.

Thank you for submitting your work to PLOS One and supporting open access.

Kind regards,

PLOS One Editorial Office Staff

on behalf of

Dr. Alpen Ortug

Academic Editor

PLOS One
